# Dynamical behavior of chaos, bifurcation analysis and soliton solutions to a Konno-Onno model

**DOI:** 10.1371/journal.pone.0291197

**Published:** 2023-09-14

**Authors:** Younes Chahlaoui, Asghar Ali, Jamshad Ahmad, Sara Javed

**Affiliations:** 1 Department of Mathematics, College of Science, King Khalid University, Abha, Saudi Arabia; 2 Department of Mathematics, Mirpur University of Science and Technology, (MUST) Mirpur, Pakistan; 3 Department of Mathematics, Faculty of Science, University of Gujrat, Gujrat, Pakistan; Institute of Space Technology, PAKISTAN

## Abstract

The fractional coupled Konno-Onno model, which is frequently used in numerous fields of scientific and engineering disciplines, is being investigated in the current study in order to gain an understanding of complex phenomena and systems. The two main goals of this study are to be accomplished. Firstly, the research aims to identify novel solitons for the fractional coupled Konno-Onno model using the unified technique, which is currently absent from the literature. Secondly, a novel strategy that hasn’t been previously investigated is phase portrait analysis for both perturbed and non-perturbed dynamical systems. The current study uses appropriate parametric values in phase plane analysis, 2D, 3D, and density plots to ensure the results are physically compatible. The results validate the claim that the technique used in this research to produce complete and uniform responses is not only simple to use and effective, but also substantially faster in computing. The technique is useful for resolving more complex phenomena that arise in engineering and mathematical physics.

## Introduction

A potent mathematical tool for modeling and comprehending complicated events characterized by memory effects, long-range interactions, and anomalous diffusion is the fractional partial differential equation (FPDE). These phenomena frequently display behaviors that conventional integer-order partial differential equations are unable to completely explain. The ability of FPDEs to include fractional derivatives, which capture the non-local and non-Markovian nature of the underlying processes, is what makes them special. FPDEs may successfully simulate systems with long-range interactions, where the influence of far-off locations or past events has a large impact on the present behavior, by introducing fractional derivatives [1–3].

They are an invaluable tool for simulating and comprehending complicated phenomena in a variety of scientific and engineering disciplines due to their adaptability and capacity to capture nuanced dynamics. Analytical solutions offer precise mathematical formulas that explain how the system will behave in relation to the relevant variables and parameters. The reliance of these solutions on the beginning conditions, boundary conditions, and system characteristics may be learned from these solutions. They provide a more in-depth investigation of the dynamics of the system by revealing the existence of singularities, critical spots, and stability characteristics of the system [4, 5]. Although numerical methods can be useful in dealing with complicated and nonlinear systems, they may not be sufficient to provide a thorough understanding of the system’s behavior. The discretization, numerical techniques, and computational parameters used in numerical solutions generally provide approximations of the results. Analytical solutions, on the other hand, offer a more thorough and accurate description of the system, giving researchers a better understanding of the phenomenon they are studying [6, 7]. The preference for analytical solutions in the study of FPDEs indicates the need for a deeper comprehension of the properties, dynamics, and underlying mechanisms of the system. Researchers can generate theoretical predictions, gain behavior insightful knowledge of the behavior of the system and direct future research in the area of fractional partial differential equations by locating clear mathematical expressions [8, 9].

In order to solve FPDEs analytically, a variety of analytical techniques were developed, including the modified F-expansion technique [10, 11], the Hirota bilinear technique [12], the kather technique [13], the modified Sardar sub-equation technique [14], the $\frac{G^{\prime }}{G}-$ expansion method [15], the direct algebraic technique [16] and many others [17–20].

The coupled Konno-Oono model (CKOS), is an integrable dispersion-less system, that was created by Konno and Oono [21]. Both CKOM’s integrability traits and its applicability to certain physical events can be credited with its significance. The system’s integrability property indicates that its quantities and symmetries are conserved. Depending on the particular system being modeled, this knowledge may have been used in optical fiber and plasma physics. The CKOM is shown as follows in [22]:

$$\begin{array}{c} \begin{array}{c} R_{xt}\left(x,t\right)-2R\left(x,t\right)M\left(x,t\right)=0,\\ M_{t}\left(x,t\right)+2R\left(x,t\right)R_{x}\left(x,t\right)=0. \end{array} \end{array}$$ (1)

The fractional form of the CKOM, i.e., the fractional coupled Konno-Onno model (FCKOM), is preferred in this study because of its advantages in mathematical modeling and optimization. Fractional derivatives provide a more accurate representation of complex physical processes and more accurately reflect system behavior. Fractional derivatives also take into consideration memory effects, in real-world systems and allow the system to retain knowledge from the past. Fractional models are more exact and precise and better suit experimental data than integer-order models. Additionally, utilizing a fractional form enables the application of cutting-edge optimization and control techniques for enhanced system performance. The following is a presentation of the mathematical form of the FCKOM [23]:

$$\begin{array}{c} \begin{array}{c} D_{xt}^{\delta \gamma }R\left(x,t\right)-2R\left(x,t\right)M\left(x,t\right)=0,\\ D_{t}^{\gamma }M\left(x,t\right)+2R\left(x,t\right)D_{x}^{\delta }R\left(x,t\right)=0, \end{array} \end{array}$$ (2)

at which 0 < *δ*, *γ* ≤ 1. The motions of both particles communicating in a medium are represented by the functions R(x,t) and M(x,t), which have fractional derivatives. Many scholars have previously addressed the CKOM and FCKOM using various analytical techniques; for example, Kocak et al. obtained the traveling wave solution for the CKOM using the modified exp-function technique [24]. The tanh-function and expanded tanh-function techniques have been used to create precise outcomes to the CKOM in [25].

The study aims to obtain a novel soliton to an FCKOM via a unified technique (UT) [26] is a novel analytical technique for solving FPDEs. Also, investigate the phase portrait analysis for the perturbed and nonperturbed dynamical system which is obtained by the nonlinear ordinary differential equation (NODE) of FCKOM. This study explores the fascinating area of chaotic behavior in nonlinear systems with the goal of identifying, describing and comprehending the resulting dynamics. The uniqueness of the research, sophisticated analytical methods, and multidisciplinary approach ensure significant contributions to fundamental science and useful applications in numerous fields. Through this work, we uncover new complexities that are at the core of nonlinear dynamical systems, enhancing our understanding of chaotic occurrences and their broader consequences. It applies variable transformations to convert FPDEs into NODEs by using conformable derivatives and then assumes a series-form solution to convert these NODEs into a system of algebraic equations. The families of soliton (also known as solitary wave) solutions for FPDEs are then found by solving the given system of algebraic equations. A soliton is a self-sustaining wave that maintains its shape and speed while neither dissipating nor spreading out. It interacts with other solitons while preserving its distinctiveness and demonstrating stability. Because nonlinearity and dispersion are carefully balanced, solitons exhibit unique properties. To better understand wave behavior, nonlinear dynamics, and integrable systems, they are studied in a variety of fields. They are useful in biological modeling, waterways, and communication networks. Solitons’ solutions give insight into how systems work and into the underlying physical processes.

The whole of the work is divided into the following sections. Section, includes the description of the conformable derivative. Section, includes the methodology of the UT. Section, includes the application of the used technique to governing model. The phase portrait analysis is provided in Section. Section, covers the results and discussions. Section, includes the conclusion and future work.

## Conformable derivative

The conformable derivative provides an alternative approach to fractional differentiation, which uses the Caputo and Riemann-Liouville procedures [27, 28]. The conformable derivative has been used in physics, engineering, and data analysis, among other fields. It has been used to represent anomalous diffusion processes, process signals, solve fractional differential equations, and analyze time series. It is significant to remember that the conformable derivative is a relatively recent addition to fractional calculus and that research into its characteristics and applications is still ongoing. A new perspective on fractional differentiation is offered, opening up new possibilities for research and understanding complex processes and systems that display fractional dynamics.

***Definition***: Let *p* : [0, ∞) → *Q*, 0 < *δ* ≤ 1 and ∀ *t* ≥ 0. The conformable derivatives of *p* order ′*δ*′ is

$$\begin{array}{c} W_{\delta }\left(p\right)\left(t\right)=\underset{\varepsilon \to 0}{\text{lim}}\;\frac{p\left(t+\varepsilon t^{1-\delta }\right)-p\left(t\right)}{\varepsilon }. \end{array}$$ (3)

***Theorem***: Assume *p*, *q* : (0, ∞) → *Q* and *δ* be a differentiable function, then chain rule holds [29]

$$\begin{array}{c} W_{\delta }\left(p\;\circ \;q\right)\left(t\right)=t^{1-\delta }q{\left(t\right)}^{\delta -2}q^{\prime }\left(t\right)W_{\delta }{\left(p\left(t\right)\right)|}_{t=q(t)}. \end{array}$$ (4)

## Methodology

In order to solve a nonlinear FPDE, the unified technique is used. The UT simplifies the process of finding solutions by providing a consistent approach to addressing multiple types of equations, making it an invaluable tool for researchers in diverse scientific and engineering domains. In numerous instances, the approach has been effectively used to solve fractional nonlinear partial differential equations in physics and mathematics.

Let’s assume the general form of nonlinear FPDEs:

$$\begin{array}{c} W(v,\partial_{t}^{\delta }v,\partial_{x}^{\gamma }v,v\partial_{xt}^{\delta }\gamma v,\ldots )=0, \end{array}$$ (5)

where 0 < *δ*, *γ* ≤ 1, *v* = *v*(*x*, *t*) is complex valued function and *x* is spatial and *t* is temporal term.

**Step 1**. Assume the transformation of waves are:

$$\begin{array}{c} R\left(x,t\right)=P\left(\Psi \right)\;\;\;M\left(x,t\right)=Q\left(\Psi \right)\;\;\Psi =s(\frac{x^{\delta }}{\delta }-q\frac{t^{\gamma }}{\gamma }), \end{array}$$ (6)

such as *s*, *q* are constant and *δ*, *γ* are fractional operator.

Putting the Eq (6) into the Eq (4), the resulting is a nonlinear ODE as:

$$\begin{array}{c} T(J\left(\Psi \right),J^{\prime }\left(\Psi \right),J^{\prime \prime }\left(\Psi \right),\ldots )=0. \end{array}$$ (7)

**Step 2**. The solution of Eq (7), can be supposed as following:

$$\begin{array}{c} J\left(\Psi \right)=l_{0}+\sum_{m=1}^{M}l_{m}\left(K{\left(\Psi \right)}^{m}\right)+\sum_{m=1}^{M}h_{m}\left(K{\left(\Psi \right)}^{-m}\right), \end{array}$$ (8)

where *l*_0_, *l*_*m*_, *h*_*m*_, (*m* = 1, 2, 3, …, *M*) are constants that are (*l*_0_, *l*_*m*_, *h*_*m*_), should not be identically zero), *M* is a term that is to be balanced, by the balancing between the terms that are nonlinear including the greatest derivative and the greatest power of Eq (7) and the function *K*(Ψ) satisfy the given below differential equation:

$$\begin{array}{c} K^{\prime }\left(\Psi \right)=K{\left(\Psi \right)}^{2}+\sigma . \end{array}$$ (9)

Where *σ* is a constant. The resulting solution of Eq (9) are given below that are depends on the *σ* parameter:

**(a)**: When *σ* < 0, we get hyperbolic solutions
  $$\begin{array}{c} K_{1}\left(\Psi \right)=\frac{\sqrt{\sigma (-(h^{2}+l^{2}))}-l\sqrt{-\sigma }\;\text{cosh}\;(2\sqrt{-\sigma }\left(\tau +\Psi \right))}{h+l\;\text{sinh}\;(2\sqrt{-\sigma }\left(\tau +\Psi \right))}, \end{array}$$
  $$\begin{array}{c} K_{2}\left(\Psi \right)=\frac{-\sqrt{\sigma (-(h^{2}+l^{2}))}-l\sqrt{-\sigma }\;\text{cosh}\;(2\sqrt{-\sigma }\left(\tau +\Psi \right))}{l\;\text{sinh}\;(2\sqrt{-\sigma }\left(\tau +\Psi \right))+\sigma }, \end{array}$$
  $$\begin{array}{c} K_{3}\left(\Psi \right)=\sqrt{-\sigma }-\frac{2l\sqrt{-\sigma }}{l-\text{sinh}\;(2\sqrt{-\sigma }\left(\tau +\Psi \right))+\;\text{cosh}\;(2\sqrt{-\sigma }\left(\tau +\Psi \right))}, \end{array}$$
  $$\begin{array}{c} K_{4}\left(\Psi \right)=-\frac{2l\sqrt{-\sigma }}{l-\text{sinh}\;(2\sqrt{-\sigma }\left(\tau +\Psi \right))+\;\text{cosh}\;(2\sqrt{-\sigma }\left(\tau +\Psi \right))}-\sqrt{-\sigma }. \end{array}$$
**(b)**: When *σ* > 0, we get trigonometric solutions
  $$\begin{array}{c} K_{5}\left(\Psi \right)=\frac{\sqrt{\sigma (l^{2}-h^{2})}-l\sqrt{\sigma }\;\text{cos}\;(2\sqrt{\sigma }\left(\tau +\Psi \right))}{l\;\text{sin}\;(2\sqrt{\sigma }\left(\tau +\Psi \right))+h}, \end{array}$$
  $$\begin{array}{c} K_{6}\left(\Psi \right)=\frac{-\sqrt{\sigma (l^{2}-h^{2})}-l\sqrt{\sigma }\;\text{cos}\;(2\sqrt{\sigma }\left(\tau +\Psi \right))}{h+l\;\text{sin}\;(2\sqrt{\sigma }\left(\tau +\Psi \right))}, \end{array}$$
  $$\begin{array}{c} K_{7}\left(\Psi \right)=i\sqrt{\sigma }-\frac{2il\sqrt{\sigma }}{l-i\;\text{sin}\;(2\sqrt{\sigma }\left(\tau +\Psi \right))+\;\text{cos}\;(2\sqrt{-\sigma }\left(\tau +\Psi \right))}, \end{array}$$
  $$\begin{array}{c} K_{8}\left(\Psi \right)=\frac{2il\sqrt{\sigma }}{l-i\;\text{sin}\;(2\sqrt{\sigma }\left(\tau +\Psi \right))+\;\text{cos}\;(2\sqrt{-\sigma }\left(\tau +\Psi \right))}-i\sqrt{\sigma }. \end{array}$$
**(c)**: When *σ* = 0, we get rational solution
  $$\begin{array}{c} K_{9}\left(\Psi \right)=-\frac{1}{\tau +\Psi }. \end{array}$$

**Step 3**. Put Eq (8) and second order require derivatives of Eq (8) into Eq (7), now consider the Eq (9), the polynomial which is obtained is power of *K*(Ψ).

**Step 4**. Collect all the coefficients of the *K*(Ψ) which have the same power and furthermore equate each coefficient to zero, then the algebraic system of the equation was derived for *l*_0_, *l*_*m*_, *h*_*m*_ (*m* = 1, 2, 3, …, *M*) and *σ*.

**Step 5**. Lastly, solve the algebraic systems of equation by using Wolfram Mathematica and found the parameter values. Putting these parameter values to Eq (8), we get the solution of Eq (2).

## Implementation

Let’s assume Eq (2) to create an accurate solution. Putting the transformation Eq (6) of a traveling wave into Eq (2), with the help of Eq (4), to get the coupled NODEs are

$$\begin{array}{c} -s^{2}q-2P\left(\Psi \right)Q\left(\Psi \right)=0 \end{array}$$ (10)

$$\begin{array}{c} -sqQ^{\prime }\left(\Psi \right)+2sP\left(\Psi \right)P^{\prime }\left(\Psi \right)=0 \end{array}$$ (11)

Integrating Eq (11), with respect to Ψ, get

$$\begin{array}{c} Q\left(\Psi \right)=\frac{L+P^{2}\left(\Psi \right)}{q} \end{array}$$ (12)

Inserting Eq (12), into Eq (10), we get the NODE is

$$\begin{array}{c} {\left(sq\right)}^{2}P^{\prime \prime }\left(\Psi \right)+2P\left(\Psi \right)L+2P^{3}\left(\Psi \right)=0 \end{array}$$ (13)

where *P*(Ψ) is complex-valued function, *s*, *q*, *L* are all constants.

By using the principle of homogeneous balance, from Eq (13) we derive that *M* = 1, by a balance between the terms of *P*^3^(Ψ) and *P*″(Ψ). So that put *M* = 1 in Eq (8) and solution of Eq (8) is supposed in the form of

$$\begin{array}{c} P\left(\Psi \right)=\frac{h_{1}}{K(\Psi )}+l_{1}K\left(\Psi \right)+l_{0}. \end{array}$$ (14)

Derivate Eq (14), twice with the help of considering Eq (9), to get a polynomial in the power of *K*(Ψ). Collect all the coefficients which have the same powers of the *K*(Ψ) and after that, set each coefficient to zero. The resultant algebraic system of equation for *l*_0_, *l*_1_, *h*_1_ and *q* as follows

$$\begin{array}{c} 2h_{1}q^{2}s^{2}\sigma^{2}+2h_{1}^{3}=0,\\ +6h_{1}^{2}l_{0}+2l_{0}L=0,\\ 12h_{1}l_{1}l_{0}+2l_{0}^{3}=0,\\ 6h_{1}l_{0}^{2}+6h_{1}^{2}l_{1}++2h_{1}L+2h_{1}q^{2}\sigma s^{2}=0,\\ +(6h_{1}l_{1}^{2}+2l_{1}L+2l_{1}q^{2}\sigma s^{2}+6l_{0}^{2}l_{1})=0,\\ +(2l_{1}q^{2}s^{2}+2l_{1}^{3})+6l_{0}l_{1}^{2}=0. \end{array}$$ (15)

When solving the algebraic system of equations, we obtained the solution sets that are

**Set-1**:

$$\begin{array}{c} \{l_{0}\to 0,l_{1}\to -\frac{\sqrt{L}}{2\sqrt{\sigma }},h_{1}\to \frac{\sqrt{L}\sqrt{\sigma }}{2},q\to -\frac{i\sqrt{L}}{2s\sqrt{\sigma }}\}. \end{array}$$ (16)

**Set-2**:

$$\begin{array}{c} \{l_{0}\to 0,l_{1}\to \frac{i\sqrt{L}}{\sqrt{2}\sqrt{\sigma }},h_{1}\to \frac{i\sqrt{L}\sqrt{\sigma }}{\sqrt{2}},q\to -\frac{\sqrt{L}}{\sqrt{2}s\sqrt{\sigma }}\}. \end{array}$$ (17)

By using Set-1 of Eq (16), the solutions for Eq (2) are as follows

$$\begin{array}{c} \begin{array}{c} R_{1,1}\left(x,t\right)=-\frac{\sqrt{L}(\sqrt{\sigma (-h^{2}-l^{2})}-l\sqrt{-\sigma }\;\text{cosh}\;(2\sqrt{-\sigma }(W)))}{2\sqrt{\sigma }(h+l\;\text{sinh}\;(2\sqrt{-\sigma }(\tau +s(\frac{x^{\delta }}{\delta }+\frac{i\sqrt{L}t^{\gamma }}{2\gamma s\sqrt{\sigma }}))))}\\ +\frac{\sqrt{L}\sqrt{\sigma }(h+l\;\text{sinh}\;(2\sqrt{-\sigma }(\tau +s(\frac{x^{\delta }}{\delta }+\frac{i\sqrt{L}t^{\gamma }}{2\gamma s\sqrt{\sigma }}))))}{2(\sqrt{\sigma (-h^{2}-l^{2})}-l\sqrt{-\sigma }\;\text{cosh}\;(2\sqrt{-\sigma }(\tau +s(\frac{x^{\delta }}{\delta }+\frac{i\sqrt{L}t^{\gamma }}{2\gamma s\sqrt{\sigma }}))))}, \end{array} \end{array}$$ (18)

$$\begin{array}{c} \begin{array}{c} M_{1,1}\left(x,t\right)=\frac{1}{q}\times (L+(-\frac{\sqrt{L}(\sqrt{\sigma (-h^{2}-l^{2})}-l\sqrt{-\sigma }\;\text{cosh}\;(2\sqrt{-\sigma }(W)))}{2\sqrt{\sigma }(h+l\;\text{sinh}\;(2\sqrt{-\sigma }(\tau +s(\frac{x^{\delta }}{\delta }+\frac{i\sqrt{L}t^{\gamma }}{2\gamma s\sqrt{\sigma }}))))}\\ +\frac{\sqrt{L}\sqrt{\sigma }(h+l\;\text{sinh}\;(2\sqrt{-\sigma }(\tau +s(\frac{x^{\delta }}{\delta }+\frac{i\sqrt{L}t^{\gamma }}{2\gamma s\sqrt{\sigma }}))))}{2(\sqrt{\sigma (-h^{2}-l^{2})}-l\sqrt{-\sigma }\;\text{cosh}\;(2\sqrt{-\sigma }(\tau +s(\frac{x^{\delta }}{\delta }+\frac{i\sqrt{L}t^{\gamma }}{2\gamma s\sqrt{\sigma }}))))}{)}^{2}), \end{array} \end{array}$$ (19)

$$\begin{array}{c} \begin{array}{c} R_{1,2}\left(x,t\right)=-\frac{\sqrt{L}(-\sqrt{\sigma (-h^{2}-l^{2})}-l\sqrt{-\sigma }\;\text{cosh}\;(2\sqrt{-\sigma }(W)))}{2\sqrt{\sigma }(\sigma +l\;\text{sinh}\;(2\sqrt{-\sigma }(\tau +s(\frac{x^{\delta }}{\delta }+\frac{i\sqrt{L}t^{\gamma }}{2\gamma s\sqrt{\sigma }}))))}\\ +\frac{\sqrt{L}\sqrt{\sigma }(\sigma +l\;\text{sinh}\;(2\sqrt{-\sigma }(\tau +s(\frac{x^{\delta }}{\delta }+\frac{i\sqrt{L}t^{\gamma }}{2\gamma s\sqrt{\sigma }}))))}{2(-\sqrt{\sigma (-h^{2}-l^{2})}-l\sqrt{-\sigma }\;\text{cosh}\;(2\sqrt{-\sigma }(\tau +s(\frac{x^{\delta }}{\delta }+\frac{i\sqrt{L}t^{\gamma }}{2\gamma s\sqrt{\sigma }}))))}, \end{array} \end{array}$$ (20)

$$\begin{array}{c} \begin{array}{c} M_{1,2}\left(x,t\right)=\frac{1}{q}\times (L+(-\frac{\sqrt{L}(-\sqrt{\sigma (-h^{2}-l^{2})}-l\sqrt{-\sigma }\;\text{cosh}\;(2\sqrt{-\sigma }(W)))}{2\sqrt{\sigma }(\sigma +l\;\text{sinh}\;(2\sqrt{-\sigma }(\tau +s(\frac{x^{\delta }}{\delta }+\frac{i\sqrt{L}t^{\gamma }}{2\gamma s\sqrt{\sigma }}))))}\\ +\frac{\sqrt{L}\sqrt{\sigma }(\sigma +l\;\text{sinh}\;(2\sqrt{-\sigma }(\tau +s(\frac{x^{\delta }}{\delta }+\frac{i\sqrt{L}t^{\gamma }}{2\gamma s\sqrt{\sigma }}))))}{2(-\sqrt{\sigma (-h^{2}-l^{2})}-l\sqrt{-\sigma }\;\text{cosh}\;(2\sqrt{-\sigma }(\tau +s(\frac{x^{\delta }}{\delta }+\frac{i\sqrt{L}t^{\gamma }}{2\gamma s\sqrt{\sigma }}))))}{)}^{2}), \end{array} \end{array}$$ (21)

$$\begin{array}{c} \begin{array}{c} R_{1,3}\left(x,t\right)=\frac{\sqrt{L}\sqrt{\sigma }}{2(\sqrt{-\sigma }-\frac{2l\sqrt{-\sigma }}{l-\text{sinh}\;(2\sqrt{-\sigma }(W))+\;\text{cosh}\;(2\sqrt{-\sigma }(\tau +s(\frac{x^{\delta }}{\delta }+\frac{i\sqrt{L}t^{\gamma }}{2\gamma s\sqrt{\sigma }})))})}\\ -\frac{\sqrt{L}(\sqrt{-\sigma }-\frac{2l\sqrt{-\sigma }}{l-\text{sinh}\;(2\sqrt{-\sigma }(\tau +s(\frac{x^{\delta }}{\delta }+\frac{i\sqrt{L}t^{\gamma }}{2\gamma s\sqrt{\sigma }})))+\;\text{cosh}\;(2\sqrt{-\sigma }(\tau +s(\frac{x^{\delta }}{\delta }+\frac{i\sqrt{L}t^{\gamma }}{2\gamma s\sqrt{\sigma }})))})}{2\sqrt{\sigma }}, \end{array} \end{array}$$ (22)

$$\begin{array}{c} \begin{array}{c} M_{1,3}\left(x,t\right)=\frac{1}{q}\times (L+(\frac{\sqrt{L}\sqrt{\sigma }}{2(\sqrt{-\sigma }-\frac{2l\sqrt{-\sigma }}{l-\text{sinh}\;(2\sqrt{-\sigma }(W))+\;\text{cosh}\;(2\sqrt{-\sigma }(\tau +s(\frac{x^{\delta }}{\delta }+\frac{i\sqrt{L}t^{\gamma }}{2\gamma s\sqrt{\sigma }})))})}\\ -\frac{\sqrt{L}(\sqrt{-\sigma }-\frac{2l\sqrt{-\sigma }}{l-\text{sinh}\;(2\sqrt{-\sigma }(\tau +s(\frac{x^{\delta }}{\delta }+\frac{i\sqrt{L}t^{\gamma }}{2\gamma s\sqrt{\sigma }})))+\;\text{cosh}\;(2\sqrt{-\sigma }(\tau +s(\frac{x^{\delta }}{\delta }+\frac{i\sqrt{L}t^{\gamma }}{2\gamma s\sqrt{\sigma }})))})}{2\sqrt{\sigma }}{)}^{2}), \end{array} \end{array}$$ (23)

$$\begin{array}{c} \begin{array}{c} R_{1,4}\left(x,t\right)=\frac{\sqrt{L}\sqrt{\sigma }}{2(-\sqrt{-\sigma }-\frac{2l\sqrt{-\sigma }}{l-\text{sinh}\;(2\sqrt{-\sigma }(W))+\;\text{cosh}\;(2\sqrt{-\sigma }(\tau +s(\frac{x^{\delta }}{\delta }+\frac{i\sqrt{L}t^{\gamma }}{2\gamma s\sqrt{\sigma }})))})}\\ -\frac{\sqrt{L}(-\sqrt{-\sigma }-\frac{2l\sqrt{-\sigma }}{l-\text{sinh}\;(2\sqrt{-\sigma }(\tau +s(\frac{x^{\delta }}{\delta }+\frac{i\sqrt{L}t^{\gamma }}{2\gamma s\sqrt{\sigma }})))+\;\text{cosh}\;(2\sqrt{-\sigma }(\tau +s(\frac{x^{\delta }}{\delta }+\frac{i\sqrt{L}t^{\gamma }}{2\gamma s\sqrt{\sigma }})))})}{2\sqrt{\sigma }}, \end{array} \end{array}$$ (24)

$$\begin{array}{c} \begin{array}{c} M_{1,4}\left(x,t\right)=\frac{1}{q}\times (L+(+\frac{\sqrt{L}\sqrt{\sigma }}{2(-\sqrt{-\sigma }-\frac{2l\sqrt{-\sigma }}{l-\text{sinh}\;(2\sqrt{-\sigma }(W))+\;\text{cosh}\;(2\sqrt{-\sigma }(\tau +s(\frac{x^{\delta }}{\delta }+\frac{i\sqrt{L}t^{\gamma }}{2\gamma s\sqrt{\sigma }})))})}\\ -\frac{\sqrt{L}(-\sqrt{-\sigma }-\frac{2l\sqrt{-\sigma }}{l-\text{sinh}\;(2\sqrt{-\sigma }(\tau +s(\frac{x^{\delta }}{\delta }+\frac{i\sqrt{L}t^{\gamma }}{2\gamma s\sqrt{\sigma }})))+\;\text{cosh}\;(2\sqrt{-\sigma }(\tau +s(\frac{x^{\delta }}{\delta }+\frac{i\sqrt{L}t^{\gamma }}{2\gamma s\sqrt{\sigma }})))})}{2\sqrt{\sigma }}{)}^{2}), \end{array} \end{array}$$ (25)

$$\begin{array}{c} \begin{array}{c} R_{1,5}\left(x,t\right)=-\frac{\sqrt{L}(\sqrt{\sigma (l^{2}-h^{2})}-l\sqrt{\sigma }\;\text{cos}\;(2\sqrt{\sigma }(W)))}{2\sqrt{\sigma }(h+l\;\text{sin}\;(2\sqrt{\sigma }(\tau +s(\frac{x^{\delta }}{\delta }+\frac{i\sqrt{L}t^{\gamma }}{2\gamma s\sqrt{\sigma }}))))}\\ +\frac{\sqrt{L}\sqrt{\sigma }(h+l\;\text{sin}\;(2\sqrt{\sigma }(\tau +s(\frac{x^{\delta }}{\delta }+\frac{i\sqrt{L}t^{\gamma }}{2\gamma s\sqrt{\sigma }}))))}{2(\sqrt{\sigma (l^{2}-h^{2})}-l\sqrt{\sigma }\;\text{cos}\;(2\sqrt{\sigma }(\tau +s(\frac{x^{\delta }}{\delta }+\frac{i\sqrt{L}t^{\gamma }}{2\gamma s\sqrt{\sigma }}))))}, \end{array} \end{array}$$ (26)

$$\begin{array}{c} \begin{array}{c} M_{1,5}\left(x,t\right)=\frac{1}{q}\times (L+(-\frac{\sqrt{L}(\sqrt{\sigma (l^{2}-h^{2})}-l\sqrt{\sigma }\;\text{cos}\;(2\sqrt{\sigma }(W)))}{2\sqrt{\sigma }(h+l\;\text{sin}\;(2\sqrt{\sigma }(\tau +s(\frac{x^{\delta }}{\delta }+\frac{i\sqrt{L}t^{\gamma }}{2\gamma s\sqrt{\sigma }}))))}\\ +\frac{\sqrt{L}\sqrt{\sigma }(h+l\;\text{sin}\;(2\sqrt{\sigma }(\tau +s(\frac{x^{\delta }}{\delta }+\frac{i\sqrt{L}t^{\gamma }}{2\gamma s\sqrt{\sigma }}))))}{2(\sqrt{\sigma (l^{2}-h^{2})}-l\sqrt{\sigma }\;\text{cos}\;(2\sqrt{\sigma }(\tau +s(\frac{x^{\delta }}{\delta }+\frac{i\sqrt{L}t^{\gamma }}{2\gamma s\sqrt{\sigma }}))))}{)}^{2}), \end{array} \end{array}$$ (27)

$$\begin{array}{c} \begin{array}{c} R_{1,6}\left(x,t\right)=-\frac{\sqrt{L}(-\sqrt{\sigma (l^{2}-h^{2})}-l\sqrt{\sigma }\;\text{cos}\;(2\sqrt{\sigma }(W)))}{2\sqrt{\sigma }(h+l\;\text{sin}\;(2\sqrt{\sigma }(\tau +s(\frac{x^{\delta }}{\delta }+\frac{i\sqrt{L}t^{\gamma }}{2\gamma s\sqrt{\sigma }}))))}\\ +\frac{\sqrt{L}\sqrt{\sigma }(h+l\;\text{sin}\;(2\sqrt{\sigma }(\tau +s(\frac{x^{\delta }}{\delta }+\frac{i\sqrt{L}t^{\gamma }}{2\gamma s\sqrt{\sigma }}))))}{2(-\sqrt{\sigma (l^{2}-h^{2})}-l\sqrt{\sigma }\;\text{cos}\;(2\sqrt{\sigma }(\tau +s(\frac{x^{\delta }}{\delta }+\frac{i\sqrt{L}t^{\gamma }}{2\gamma s\sqrt{\sigma }}))))} \end{array} \end{array}$$ (28)

$$\begin{array}{c} \begin{array}{c} M_{1,6}\left(x,t\right)=\frac{1}{q}\times (L+(-\frac{\sqrt{L}(-\sqrt{\sigma (l^{2}-h^{2})}-l\sqrt{\sigma }\;\text{cos}\;(2\sqrt{\sigma }(W)))}{2\sqrt{\sigma }(h+l\;\text{sin}\;(2\sqrt{\sigma }(\tau +s(\frac{x^{\delta }}{\delta }+\frac{i\sqrt{L}t^{\gamma }}{2\gamma s\sqrt{\sigma }}))))}\\ +\frac{\sqrt{L}\sqrt{\sigma }(h+l\;\text{sin}\;(2\sqrt{\sigma }(\tau +s(\frac{x^{\delta }}{\delta }+\frac{i\sqrt{L}t^{\gamma }}{2\gamma s\sqrt{\sigma }}))))}{2(-\sqrt{\sigma (l^{2}-h^{2})}-l\sqrt{\sigma }\;\text{cos}\;(2\sqrt{\sigma }(\tau +s(\frac{x^{\delta }}{\delta }+\frac{i\sqrt{L}t^{\gamma }}{2\gamma s\sqrt{\sigma }}))))}{)}^{2}), \end{array} \end{array}$$ (29)

$$\begin{array}{c} \begin{array}{c} R_{1,7}\left(x,t\right)=\frac{\sqrt{L}\sqrt{\sigma }}{2(i\sqrt{\sigma }-\frac{2il\sqrt{\sigma }}{l-i\;\text{sin}\;(2\sqrt{\sigma }(W))+\;\text{cos}\;(2\sqrt{-\sigma }(\tau +s(\frac{x^{\delta }}{\delta }+\frac{i\sqrt{L}t^{\gamma }}{2\gamma s\sqrt{\sigma }})))})}\\ -\frac{\sqrt{L}(i\sqrt{\sigma }-\frac{2il\sqrt{\sigma }}{l-i\;\text{sin}\;(2\sqrt{\sigma }(\tau +s(\frac{x^{\delta }}{\delta }+\frac{i\sqrt{L}t^{\gamma }}{2\gamma s\sqrt{\sigma }})))+\;\text{cos}\;(2\sqrt{-\sigma }(\tau +s(\frac{x^{\delta }}{\delta }+\frac{i\sqrt{L}t^{\gamma }}{2\gamma s\sqrt{\sigma }})))})}{2\sqrt{\sigma }}, \end{array} \end{array}$$ (30)

$$\begin{array}{c} \begin{array}{c} M_{1,7}\left(x,t\right)=\frac{1}{q}\times (L+(\frac{\sqrt{L}\sqrt{\sigma }}{2(i\sqrt{\sigma }-\frac{2il\sqrt{\sigma }}{l-i\;\text{sin}\;(2\sqrt{\sigma }(W))+\;\text{cos}\;(2\sqrt{-\sigma }(\tau +s(\frac{x^{\delta }}{\delta }+\frac{i\sqrt{L}t^{\gamma }}{2\gamma s\sqrt{\sigma }})))})}\\ -\frac{\sqrt{L}(i\sqrt{\sigma }-\frac{2il\sqrt{\sigma }}{l-i\;\text{sin}\;(2\sqrt{\sigma }(\tau +s(\frac{x^{\delta }}{\delta }+\frac{i\sqrt{L}t^{\gamma }}{2\gamma s\sqrt{\sigma }})))+\;\text{cos}\;(2\sqrt{-\sigma }(\tau +s(\frac{x^{\delta }}{\delta }+\frac{i\sqrt{L}t^{\gamma }}{2\gamma s\sqrt{\sigma }})))})}{2\sqrt{\sigma }}{)}^{2}), \end{array} \end{array}$$ (31)

$$\begin{array}{c} \begin{array}{c} R_{1,8}\left(x,t\right)=\frac{\sqrt{L}\sqrt{\sigma }}{2(\frac{2il\sqrt{\sigma }}{l-i\;\text{sin}\;(2\sqrt{\sigma }(W))+\;\text{cos}\;(2\sqrt{-\sigma }(\tau +s(\frac{x^{\delta }}{\delta }+\frac{i\sqrt{L}t^{\gamma }}{2\gamma s\sqrt{\sigma }})))}-i\sqrt{\sigma })}\\ -\frac{\sqrt{L}(\frac{2il\sqrt{\sigma }}{l-i\;\text{sin}\;(2\sqrt{\sigma }(\tau +s(\frac{x^{\delta }}{\delta }+\frac{i\sqrt{L}t^{\gamma }}{2\gamma s\sqrt{\sigma }})))+\;\text{cos}\;(2\sqrt{-\sigma }(\tau +s(\frac{x^{\delta }}{\delta }+\frac{i\sqrt{L}t^{\gamma }}{2\gamma s\sqrt{\sigma }})))}-i\sqrt{\sigma })}{2\sqrt{\sigma }}, \end{array} \end{array}$$ (32)

$$\begin{array}{c} \begin{array}{c} M_{1,8}\left(x,t\right)=\frac{1}{q}\times (L+(\frac{\sqrt{L}\sqrt{\sigma }}{2(\frac{2il\sqrt{\sigma }}{l-i\;\text{sin}\;(2\sqrt{\sigma }(W))+\;\text{cos}\;(2\sqrt{-\sigma }(\tau +s(\frac{x^{\delta }}{\delta }+\frac{i\sqrt{L}t^{\gamma }}{2\gamma s\sqrt{\sigma }})))}-i\sqrt{\sigma })}\\ -\frac{\sqrt{L}(\frac{2il\sqrt{\sigma }}{l-i\;\text{sin}\;(2\sqrt{\sigma }(\tau +s(\frac{x^{\delta }}{\delta }+\frac{i\sqrt{L}t^{\gamma }}{2\gamma s\sqrt{\sigma }})))+\;\text{cos}\;(2\sqrt{-\sigma }(\tau +s(\frac{x^{\delta }}{\delta }+\frac{i\sqrt{L}t^{\gamma }}{2\gamma s\sqrt{\sigma }})))}-i\sqrt{\sigma })}{2\sqrt{\sigma }}{)}^{2}), \end{array} \end{array}$$ (33)

$$\begin{array}{c} \begin{array}{c} R_{1,9}\left(x,t\right)=\frac{1}{2}\sqrt{L}\sqrt{\sigma }(-\tau -s(\frac{x^{\delta }}{\delta }+\frac{i\sqrt{L}t^{\gamma }}{2\gamma s\sqrt{\sigma }}))+\frac{\sqrt{L}}{2\sqrt{\sigma }(\tau +s(\frac{x^{\delta }}{\delta }+\frac{i\sqrt{L}t^{\gamma }}{2\gamma s\sqrt{\sigma }}))} \end{array} \end{array}$$ (34)

$$\begin{array}{c} \begin{array}{c} M_{1,9}\left(x,t\right)=\frac{1}{q}\times (L+{\left(\frac{1}{2}\sqrt{L}\sqrt{\sigma }(-W)+\frac{\sqrt{L}}{2\sqrt{\sigma }(\tau +s(\frac{x^{\delta }}{\delta }+\frac{i\sqrt{L}t^{\gamma }}{2\gamma s\sqrt{\sigma }}))}\right)}^{2}). \end{array} \end{array}$$ (35)

By using Set-2 of Eq (17), the solutions for Eq (2) are as follows

$$\begin{array}{c} \begin{array}{c} R_{2,1}\left(x,t\right)=\frac{i\sqrt{L}(\sqrt{\sigma (-h^{2}-l^{2})}-l\sqrt{-\sigma }\;\text{cosh}\;(2\sqrt{-\sigma }(W)))}{\sqrt{2}\sqrt{\sigma }(h+l\;\text{sinh}\;(2\sqrt{-\sigma }(s(\frac{\sqrt{L}t^{\gamma }}{\sqrt{2}\gamma s\sqrt{\sigma }}+\frac{x^{\delta }}{\delta })+\tau )))}\\ +\frac{i\sqrt{L}\sqrt{\sigma }(h+l\;\text{sinh}\;(2\sqrt{-\sigma }(s(\frac{\sqrt{L}t^{\gamma }}{\sqrt{2}\gamma s\sqrt{\sigma }}+\frac{x^{\delta }}{\delta })+\tau )))}{\sqrt{2}(\sqrt{\sigma (-h^{2}-l^{2})}-l\sqrt{-\sigma }\;\text{cosh}\;(2\sqrt{-\sigma }(s(\frac{\sqrt{L}t^{\gamma }}{\sqrt{2}\gamma s\sqrt{\sigma }}+\frac{x^{\delta }}{\delta })+\tau )))}, \end{array} \end{array}$$ (36)

$$\begin{array}{c} \begin{array}{c} M_{2,1}\left(x,t\right)=\frac{1}{q}\times (L+(\frac{i\sqrt{L}(\sqrt{\sigma (-h^{2}-l^{2})}-l\sqrt{-\sigma }\;\text{cosh}\;(2\sqrt{-\sigma }(W)))}{\sqrt{2}\sqrt{\sigma }(h+l\;\text{sinh}\;(2\sqrt{-\sigma }(s(\frac{\sqrt{L}t^{\gamma }}{\sqrt{2}\gamma s\sqrt{\sigma }}+\frac{x^{\delta }}{\delta })+\tau )))}\\ +\frac{i\sqrt{L}\sqrt{\sigma }(h+l\;\text{sinh}\;(2\sqrt{-\sigma }(s(\frac{\sqrt{L}t^{\gamma }}{\sqrt{2}\gamma s\sqrt{\sigma }}+\frac{x^{\delta }}{\delta })+\tau )))}{\sqrt{2}(\sqrt{\sigma (-h^{2}-l^{2})}-l\sqrt{-\sigma }\;\text{cosh}\;(2\sqrt{-\sigma }(s(\frac{\sqrt{L}t^{\gamma }}{\sqrt{2}\gamma s\sqrt{\sigma }}+\frac{x^{\delta }}{\delta })+\tau )))}{)}^{2}), \end{array} \end{array}$$ (37)

$$\begin{array}{c} \begin{array}{c} R_{2,2}\left(x,t\right)=\frac{i\sqrt{L}(-\sqrt{\sigma (-h^{2}-l^{2})}-l\sqrt{-\sigma }\;\text{cosh}\;(2\sqrt{-\sigma }(W)))}{\sqrt{2}\sqrt{\sigma }(l\;\text{sinh}\;(2\sqrt{-\sigma }(s(\frac{\sqrt{L}t^{\gamma }}{\sqrt{2}\gamma s\sqrt{\sigma }}+\frac{x^{\delta }}{\delta })+\tau ))+\sigma )}\\ +\frac{i\sqrt{L}\sqrt{\sigma }(l\;\text{sinh}\;(2\sqrt{-\sigma }(s(\frac{\sqrt{L}t^{\gamma }}{\sqrt{2}\gamma s\sqrt{\sigma }}+\frac{x^{\delta }}{\delta })+\tau ))+\sigma )}{\sqrt{2}(-\sqrt{\sigma (-h^{2}-l^{2})}-l\sqrt{-\sigma }\;\text{cosh}\;(2\sqrt{-\sigma }(s(\frac{\sqrt{L}t^{\gamma }}{\sqrt{2}\gamma s\sqrt{\sigma }}+\frac{x^{\delta }}{\delta })+\tau )))}, \end{array} \end{array}$$ (38)

$$\begin{array}{c} \begin{array}{c} M_{2,2}\left(x,t\right)=\frac{1}{q}\times (L+(\frac{i\sqrt{L}(-\sqrt{\sigma (-h^{2}-l^{2})}-l\sqrt{-\sigma }\;\text{cosh}\;(2\sqrt{-\sigma }(W)))}{\sqrt{2}\sqrt{\sigma }(l\;\text{sinh}\;(2\sqrt{-\sigma }(s(\frac{\sqrt{L}t^{\gamma }}{\sqrt{2}\gamma s\sqrt{\sigma }}+\frac{x^{\delta }}{\delta })+\tau ))+\sigma )}\\ +\frac{i\sqrt{L}\sqrt{\sigma }(l\;\text{sinh}\;(2\sqrt{-\sigma }(s(\frac{\sqrt{L}t^{\gamma }}{\sqrt{2}\gamma s\sqrt{\sigma }}+\frac{x^{\delta }}{\delta })+\tau ))+\sigma )}{\sqrt{2}(-\sqrt{\sigma (-h^{2}-l^{2})}-l\sqrt{-\sigma }\;\text{cosh}\;(2\sqrt{-\sigma }(s(\frac{\sqrt{L}t^{\gamma }}{\sqrt{2}\gamma s\sqrt{\sigma }}+\frac{x^{\delta }}{\delta })+\tau )))}{)}^{2}), \end{array} \end{array}$$ (39)

$$\begin{array}{c} \begin{array}{c} R_{2,3}\left(x,t\right)=\frac{i\sqrt{L}\sqrt{\sigma }}{\sqrt{2}(\sqrt{-\sigma }-\frac{2l\sqrt{-\sigma }}{l-\text{sinh}\;(2\sqrt{-\sigma }(W))+\;\text{cosh}\;(2\sqrt{-\sigma }(s(\frac{\sqrt{L}t^{\gamma }}{\sqrt{2}\gamma s\sqrt{\sigma }}+\frac{x^{\delta }}{\delta })+\tau ))})}\\ +\frac{i\sqrt{L}(\sqrt{-\sigma }-\frac{2l\sqrt{-\sigma }}{l-\text{sinh}\;(2\sqrt{-\sigma }(s(\frac{\sqrt{L}t^{\gamma }}{\sqrt{2}\gamma s\sqrt{\sigma }}+\frac{x^{\delta }}{\delta })+\tau ))+\;\text{cosh}\;(2\sqrt{-\sigma }(s(\frac{\sqrt{L}t^{\gamma }}{\sqrt{2}\gamma s\sqrt{\sigma }}+\frac{x^{\delta }}{\delta })+\tau ))})}{\sqrt{2}\sqrt{\sigma }}, \end{array} \end{array}$$ (40)

$$\begin{array}{c} \begin{array}{c} M_{2,3}\left(x,t\right)=\frac{1}{q}\times (L+(\frac{i\sqrt{L}\sqrt{\sigma }}{\sqrt{2}(\sqrt{-\sigma }-\frac{2l\sqrt{-\sigma }}{l-\text{sinh}\;(2\sqrt{-\sigma }(W))+\;\text{cosh}\;(2\sqrt{-\sigma }(s(\frac{\sqrt{L}t^{\gamma }}{\sqrt{2}\gamma s\sqrt{\sigma }}+\frac{x^{\delta }}{\delta })+\tau ))})}\\ +\frac{i\sqrt{L}(\sqrt{-\sigma }-\frac{2l\sqrt{-\sigma }}{l-\text{sinh}\;(2\sqrt{-\sigma }(s(\frac{\sqrt{L}t^{\gamma }}{\sqrt{2}\gamma s\sqrt{\sigma }}+\frac{x^{\delta }}{\delta })+\tau ))+\;\text{cosh}\;(2\sqrt{-\sigma }(s(\frac{\sqrt{L}t^{\gamma }}{\sqrt{2}\gamma s\sqrt{\sigma }}+\frac{x^{\delta }}{\delta })+\tau ))})}{\sqrt{2}\sqrt{\sigma }}{)}^{2}), \end{array} \end{array}$$ (41)

$$\begin{array}{c} \begin{array}{c} R_{2,4}\left(x,t\right)=\frac{i\sqrt{L}\sqrt{\sigma }}{\sqrt{2}(-\frac{2l\sqrt{-\sigma }}{l-\text{sinh}\;(2\sqrt{-\sigma }(W))+\;\text{cosh}\;(2\sqrt{-\sigma }(s(\frac{\sqrt{L}t^{\gamma }}{\sqrt{2}\gamma s\sqrt{\sigma }}+\frac{x^{\delta }}{\delta })+\tau ))}-\sqrt{-\sigma })}\\ +\frac{i\sqrt{L}(-\frac{2l\sqrt{-\sigma }}{l-\text{sinh}\;(2\sqrt{-\sigma }(s(\frac{\sqrt{L}t^{\gamma }}{\sqrt{2}\gamma s\sqrt{\sigma }}+\frac{x^{\delta }}{\delta })+\tau ))+\;\text{cosh}\;(2\sqrt{-\sigma }(s(\frac{\sqrt{L}t^{\gamma }}{\sqrt{2}\gamma s\sqrt{\sigma }}+\frac{x^{\delta }}{\delta })+\tau ))}-\sqrt{-\sigma })}{\sqrt{2}\sqrt{\sigma }}, \end{array} \end{array}$$ (42)

$$\begin{array}{c} \begin{array}{c} M_{2,4}\left(x,t\right)=\frac{1}{q}\times (L+(\frac{i\sqrt{L}\sqrt{\sigma }}{\sqrt{2}(-\frac{2l\sqrt{-\sigma }}{l-\text{sinh}\;(2\sqrt{-\sigma }(W))+\;\text{cosh}\;(2\sqrt{-\sigma }(s(\frac{\sqrt{L}t^{\gamma }}{\sqrt{2}\gamma s\sqrt{\sigma }}+\frac{x^{\delta }}{\delta })+\tau ))}-\sqrt{-\sigma })}\\ +\frac{i\sqrt{L}(-\frac{2l\sqrt{-\sigma }}{l-\text{sinh}\;(2\sqrt{-\sigma }(s(\frac{\sqrt{L}t^{\gamma }}{\sqrt{2}\gamma s\sqrt{\sigma }}+\frac{x^{\delta }}{\delta })+\tau ))+\;\text{cosh}\;(2\sqrt{-\sigma }(s(\frac{\sqrt{L}t^{\gamma }}{\sqrt{2}\gamma s\sqrt{\sigma }}+\frac{x^{\delta }}{\delta })+\tau ))}-\sqrt{-\sigma })}{\sqrt{2}\sqrt{\sigma }}{)}^{2}), \end{array} \end{array}$$ (43)

$$\begin{array}{c} \begin{array}{c} R_{2,5}\left(x,t\right)=\frac{i\sqrt{L}(\sqrt{\sigma (l^{2}-h^{2})}-l\sqrt{\sigma }\;\text{cos}\;(2\sqrt{\sigma }(W)))}{\sqrt{2}\sqrt{\sigma }(l\;\text{sin}\;(2\sqrt{\sigma }(s(\frac{\sqrt{L}t^{\gamma }}{\sqrt{2}\gamma s\sqrt{\sigma }}+\frac{x^{\delta }}{\delta })+\tau ))+h)}\\ +\frac{i\sqrt{L}\sqrt{\sigma }(l\;\text{sin}\;(2\sqrt{\sigma }(s(\frac{\sqrt{L}t^{\gamma }}{\sqrt{2}\gamma s\sqrt{\sigma }}+\frac{x^{\delta }}{\delta })+\tau ))+h)}{\sqrt{2}(\sqrt{\sigma (l^{2}-h^{2})}-l\sqrt{\sigma }\;\text{cos}\;(2\sqrt{\sigma }(s(\frac{\sqrt{L}t^{\gamma }}{\sqrt{2}\gamma s\sqrt{\sigma }}+\frac{x^{\delta }}{\delta })+\tau )))}, \end{array} \end{array}$$ (44)

$$\begin{array}{c} \begin{array}{c} M_{2,5}\left(x,t\right)=\frac{1}{q}\times (L+(\frac{i\sqrt{L}(\sqrt{\sigma (l^{2}-h^{2})}-l\sqrt{\sigma }\;\text{cos}\;(2\sqrt{\sigma }(W)))}{\sqrt{2}\sqrt{\sigma }(l\;\text{sin}\;(2\sqrt{\sigma }(s(\frac{\sqrt{L}t^{\gamma }}{\sqrt{2}\gamma s\sqrt{\sigma }}+\frac{x^{\delta }}{\delta })+\tau ))+h)}\\ +\frac{i\sqrt{L}\sqrt{\sigma }(l\;\text{sin}\;(2\sqrt{\sigma }(s(\frac{\sqrt{L}t^{\gamma }}{\sqrt{2}\gamma s\sqrt{\sigma }}+\frac{x^{\delta }}{\delta })+\tau ))+h)}{\sqrt{2}(\sqrt{\sigma (l^{2}-h^{2})}-l\sqrt{\sigma }\;\text{cos}\;(2\sqrt{\sigma }(s(\frac{\sqrt{L}t^{\gamma }}{\sqrt{2}\gamma s\sqrt{\sigma }}+\frac{x^{\delta }}{\delta })+\tau )))}{)}^{2}), \end{array} \end{array}$$ (45)

$$\begin{array}{c} \begin{array}{c} R_{2,6}\left(x,t\right)=\frac{i\sqrt{L}(-\sqrt{\sigma (l^{2}-h^{2})}-l\sqrt{\sigma }\;\text{cos}\;(2\sqrt{\sigma }(W)))}{\sqrt{2}\sqrt{\sigma }(h+l\;\text{sin}\;(2\sqrt{\sigma }(s(\frac{\sqrt{L}t^{\gamma }}{\sqrt{2}\gamma s\sqrt{\sigma }}+\frac{x^{\delta }}{\delta })+\tau )))}\\ +\frac{i\sqrt{L}\sqrt{\sigma }(h+l\;\text{sin}\;(2\sqrt{\sigma }(s(\frac{\sqrt{L}t^{\gamma }}{\sqrt{2}\gamma s\sqrt{\sigma }}+\frac{x^{\delta }}{\delta })+\tau )))}{\sqrt{2}(-\sqrt{\sigma (l^{2}-h^{2})}-l\sqrt{\sigma }\;\text{cos}\;(2\sqrt{\sigma }(s(\frac{\sqrt{L}t^{\gamma }}{\sqrt{2}\gamma s\sqrt{\sigma }}+\frac{x^{\delta }}{\delta })+\tau )))}, \end{array} \end{array}$$ (46)

$$\begin{array}{c} \begin{array}{c} M_{2,6}\left(x,t\right)=\frac{1}{q}\times (L+(\frac{i\sqrt{L}(-\sqrt{\sigma (l^{2}-h^{2})}-l\sqrt{\sigma }\;\text{cos}\;(2\sqrt{\sigma }(W)))}{\sqrt{2}\sqrt{\sigma }(h+l\;\text{sin}\;(2\sqrt{\sigma }(s(\frac{\sqrt{L}t^{\gamma }}{\sqrt{2}\gamma s\sqrt{\sigma }}+\frac{x^{\delta }}{\delta })+\tau )))}\\ +\frac{i\sqrt{L}\sqrt{\sigma }(h+l\;\text{sin}\;(2\sqrt{\sigma }(s(\frac{\sqrt{L}t^{\gamma }}{\sqrt{2}\gamma s\sqrt{\sigma }}+\frac{x^{\delta }}{\delta })+\tau )))}{\sqrt{2}(-\sqrt{\sigma (l^{2}-h^{2})}-l\sqrt{\sigma }\;\text{cos}\;(2\sqrt{\sigma }(s(\frac{\sqrt{L}t^{\gamma }}{\sqrt{2}\gamma s\sqrt{\sigma }}+\frac{x^{\delta }}{\delta })+\tau )))}{)}^{2}), \end{array} \end{array}$$ (47)

$$\begin{array}{c} \begin{array}{c} R_{2,7}\left(x,t\right)=\frac{i\sqrt{L}\sqrt{\sigma }}{\sqrt{2}(i\sqrt{\sigma }-\frac{2il\sqrt{\sigma }}{l-i\;\text{sin}\;(2\sqrt{\sigma }(W))+\;\text{cos}\;(2\sqrt{-\sigma }(s(\frac{\sqrt{L}t^{\gamma }}{\sqrt{2}\gamma s\sqrt{\sigma }}+\frac{x^{\delta }}{\delta })+\tau ))})}\\ +\frac{i\sqrt{L}(i\sqrt{\sigma }-\frac{2il\sqrt{\sigma }}{l-i\;\text{sin}\;(2\sqrt{\sigma }(s(\frac{\sqrt{L}t^{\gamma }}{\sqrt{2}\gamma s\sqrt{\sigma }}+\frac{x^{\delta }}{\delta })+\tau ))+\;\text{cos}\;(2\sqrt{-\sigma }(s(\frac{\sqrt{L}t^{\gamma }}{\sqrt{2}\gamma s\sqrt{\sigma }}+\frac{x^{\delta }}{\delta })+\tau ))})}{\sqrt{2}\sqrt{\sigma }}, \end{array} \end{array}$$ (48)

$$\begin{array}{c} \begin{array}{c} M_{2,7}\left(x,t\right)=\frac{1}{q}\times (L+(\frac{i\sqrt{L}\sqrt{\sigma }}{\sqrt{2}(i\sqrt{\sigma }-\frac{2il\sqrt{\sigma }}{l-i\;\text{sin}\;(2\sqrt{\sigma }(W))+\;\text{cos}\;(2\sqrt{-\sigma }(s(\frac{\sqrt{L}t^{\gamma }}{\sqrt{2}\gamma s\sqrt{\sigma }}+\frac{x^{\delta }}{\delta })+\tau ))})}\\ +\frac{i\sqrt{L}(i\sqrt{\sigma }-\frac{2il\sqrt{\sigma }}{l-i\;\text{sin}\;(2\sqrt{\sigma }(s(\frac{\sqrt{L}t^{\gamma }}{\sqrt{2}\gamma s\sqrt{\sigma }}+\frac{x^{\delta }}{\delta })+\tau ))+\;\text{cos}\;(2\sqrt{-\sigma }(s(\frac{\sqrt{L}t^{\gamma }}{\sqrt{2}\gamma s\sqrt{\sigma }}+\frac{x^{\delta }}{\delta })+\tau ))})}{\sqrt{2}\sqrt{\sigma }}{)}^{2}), \end{array} \end{array}$$ (49)

$$\begin{array}{c} \begin{array}{c} R_{2,8}\left(x,t\right)=\frac{i\sqrt{L}\sqrt{\sigma }}{\sqrt{2}(\frac{2il\sqrt{\sigma }}{l-i\;\text{sin}\;(2\sqrt{\sigma }(W))+\;\text{cos}\;(2\sqrt{-\sigma }(s(\frac{\sqrt{L}t^{\gamma }}{\sqrt{2}\gamma s\sqrt{\sigma }}+\frac{x^{\delta }}{\delta })+\tau ))}-i\sqrt{\sigma })}\\ +\frac{i\sqrt{L}(\frac{2il\sqrt{\sigma }}{l-i\;\text{sin}\;(2\sqrt{\sigma }(s(\frac{\sqrt{L}t^{\gamma }}{\sqrt{2}\gamma s\sqrt{\sigma }}+\frac{x^{\delta }}{\delta })+\tau ))+\;\text{cos}\;(2\sqrt{-\sigma }(s(\frac{\sqrt{L}t^{\gamma }}{\sqrt{2}\gamma s\sqrt{\sigma }}+\frac{x^{\delta }}{\delta })+\tau ))}-i\sqrt{\sigma })}{\sqrt{2}\sqrt{\sigma }}, \end{array} \end{array}$$ (50)

$$\begin{array}{c} \begin{array}{c} M_{2,8}\left(x,t\right)=\frac{1}{q}\times (L+(\frac{i\sqrt{L}\sqrt{\sigma }}{\sqrt{2}(\frac{2il\sqrt{\sigma }}{l-i\;\text{sin}\;(2\sqrt{\sigma }(W))+\;\text{cos}\;(2\sqrt{-\sigma }(s(\frac{\sqrt{L}t^{\gamma }}{\sqrt{2}\gamma s\sqrt{\sigma }}+\frac{x^{\delta }}{\delta })+\tau ))}-i\sqrt{\sigma })}\\ +\frac{i\sqrt{L}(\frac{2il\sqrt{\sigma }}{l-i\;\text{sin}\;(2\sqrt{\sigma }(s(\frac{\sqrt{L}t^{\gamma }}{\sqrt{2}\gamma s\sqrt{\sigma }}+\frac{x^{\delta }}{\delta })+\tau ))+\;\text{cos}\;(2\sqrt{-\sigma }(s(\frac{\sqrt{L}t^{\gamma }}{\sqrt{2}\gamma s\sqrt{\sigma }}+\frac{x^{\delta }}{\delta })+\tau ))}-i\sqrt{\sigma })}{\sqrt{2}\sqrt{\sigma }}{)}^{2}), \end{array} \end{array}$$ (51)

$$\begin{array}{c} \begin{array}{c} R_{2,9}\left(x,t\right)=\frac{i\sqrt{L}\sqrt{\sigma }(-s(\frac{\sqrt{L}t^{\gamma }}{\sqrt{2}\gamma s\sqrt{\sigma }}+\frac{x^{\delta }}{\delta })-\tau )}{\sqrt{2}}-\frac{i\sqrt{L}}{\sqrt{2}\sqrt{\sigma }(s(\frac{\sqrt{L}t^{\gamma }}{\sqrt{2}\gamma s\sqrt{\sigma }}+\frac{x^{\delta }}{\delta })+\tau )}, \end{array} \end{array}$$ (52)

$$\begin{array}{c} \begin{array}{c} M_{2,9}\left(x,t\right)=\frac{1}{q}\times (L+{\left(\frac{i\sqrt{L}\sqrt{\sigma }(-W)}{\sqrt{2}}-\frac{i\sqrt{L}}{\sqrt{2}\sqrt{\sigma }(s(\frac{\sqrt{L}t^{\gamma }}{\sqrt{2}\gamma s\sqrt{\sigma }}+\frac{x^{\delta }}{\delta })+\tau )}\right)}^{2}). \end{array} \end{array}$$ (53)

Where from Eqs (18)–(53), $W=\tau +s(\frac{x^{\delta }}{\delta }+\frac{i\sqrt{-L}t^{\gamma }}{2\gamma s\sqrt{-\sigma }})$.

## Phase portrait analysis

A mathematical method called phase portrait analysis is used to examine the behavior of dynamic systems. Plotting a series of trajectories on the phase plane, each of which stands for a potential state of the system [30]. A dynamical system’s behavior can be very dependent on its initial characteristics and conditions, which can result in a variety of phase depictions and trajectory types. Dynamical systems are capable of experiencing events like bifurcation and chaos. Understanding the underlying dynamics and causes of complex systems through the analysis of bifurcations and chaotic behavior aids in deciphering their complex behaviors [31]. Following is the phase portrait analysis of the dynamical system for perturbed and nonperturbed systems.

### Bifurcation

There are numerous kinds of system bifurcations that can occur. On the dynamics and stability of the system, respectively. Use bifurcation diagrams to see how distinct dynamic regimes transition from one into another. the areas of parameter space where bifurcations take place and the effects they have on how the system behaves. Finding out if there are any higher-order bifurcations will give you a fuller understanding of the system’s complexity.

### Chaos

An intriguing phenomenon known as chaos can be found in some nonlinear dynamical systems. Even though the system’s basic equations are deterministic and clearly specified, it is characterized by sensitive dependency on initial conditions, which causes unexpected and apparently random behavior over time. Numerous scientific fields, including physics, mathematics, biology, engineering, and economics, have conducted substantial research on chaos. Here are a few of chaos’ main characteristics.

### Nonperturbed dynamical system

Understanding the qualitative variations in such systems’ behavior a parameter or set of parameters is changed is the main objective of bifurcation theory. A nonperturbed dynamical system can exhibit qualitative variations in behavior, and the fundamental mechanisms regulating these changes can be revealed by applying bifurcation theory to the system [32]. This analysis contributes to a deeper comprehension of the system’s behavior and prospective applications by demonstrating how the system switches between various dynamical regimes as the parameter(s) change. Using bifurcation theory [33], we shall analyze Eq (2) in this section. It is possible to examine Eq (13) as a planar dynamical system by applying a Galilean transformation.

$$\left\{ \begin{array}{c} \;\;\;\;\frac{dP}{d\Psi }=U\\ \frac{dU}{d\Psi }=-2f_{1}\left(P^{3}\right)-2f_{2}P=V \end{array}\right.$$ (54)

where $f_{1}=\frac{1}{{\left(sq\right)}^{2}}$ and $f_{2}=\frac{L}{{\left(sq\right)}^{2}}$ such that *s*, *q* and *L* are parameters. We will now talk about the bifurcations of the phase profiles of system Eq (54), which occur in the parameter space *f*_1_ and *f*_2_. These are the outcomes of our qualitative analysis. First of all, take note that system Eq (54) has three equilibrium points, which are as follows:

$$\begin{array}{c} \xi_{1}=\left(0,0\right),\;\;\;\;\;\;\xi_{2}=\left(\sqrt{\frac{-f_{2}}{f_{1}}},0\right),\;\;\;\;\;\;\xi_{3}=\left(-\sqrt{\frac{-f_{2}}{f_{1}}},0\right) \end{array}$$

Moreover, the system’s Jacobian is:

$$\begin{array}{c} J\left(U,V\right)=|\begin{array}{cc} 0 & 1\\ -2f_{2}-6f_{1}P^{2} & 0 \end{array}|=+2f_{2}+6f_{1}P^{2}. \end{array}$$ (55)

Hence, (*U*, 0) is a saddle for **J**(*U*, *V*)) < 0, (*U*, 0) is a center for **J**(*U*, *V*) > 0 and a (*U*, 0) is a cuspidal for **J**(*U*, *V*) = 0.

The following results are potential changes in the parameter values involved.

- **Case-(4.1)** When *f*_1_ < 0 & *f*_2_ < 0, Eq (54) provides three equilibrium points, *Y*_1_ = (0, 0), *Y*_2_ = (1, 0) & *Y*_3_ = (−1, 0); since *Y*_1_, *Y*_2_ & *Y*_3_ are cuspidal points as shown in Fig 15.
- **Case-(4.2)** When *f*_1_ > 0 & *f*_2_ < 0, Eq (54) provides three equilibrium points, *Y*_1_ = (0, 0), *Y*_2_ = (1, 0) & *Y*_3_ = (−1, 0); since *Y*_1_, *Y*_2_ & *Y*_3_ are center points as shown in Fig 16.
- **Case-(4.3)** When *f*_1_ < 0 & *f*_2_ > 0, Eq (54) provides three equilibrium points, *Y*_1_ = (0, 0), *Y*_2_ = (1, 0) & *Y*_3_ = (−1, 0); since *Y*_1_, *Y*_2_ & *Y*_3_ are saddle points as shown in Fig 17.
- **Case-(4.4)** When *f*_1_ > 0 & *f*_2_ > 0, Eq (54) provides three equilibrium points, *Y*_1_ = (0, 0), *Y*_2_ = (1, 0) & *Y*_3_ = (−1, 0); since *Y*_1_, & *Y*_2_ are saddle points and *Y*_3_ is center point as shown in Fig 18.

**Fig 1:**
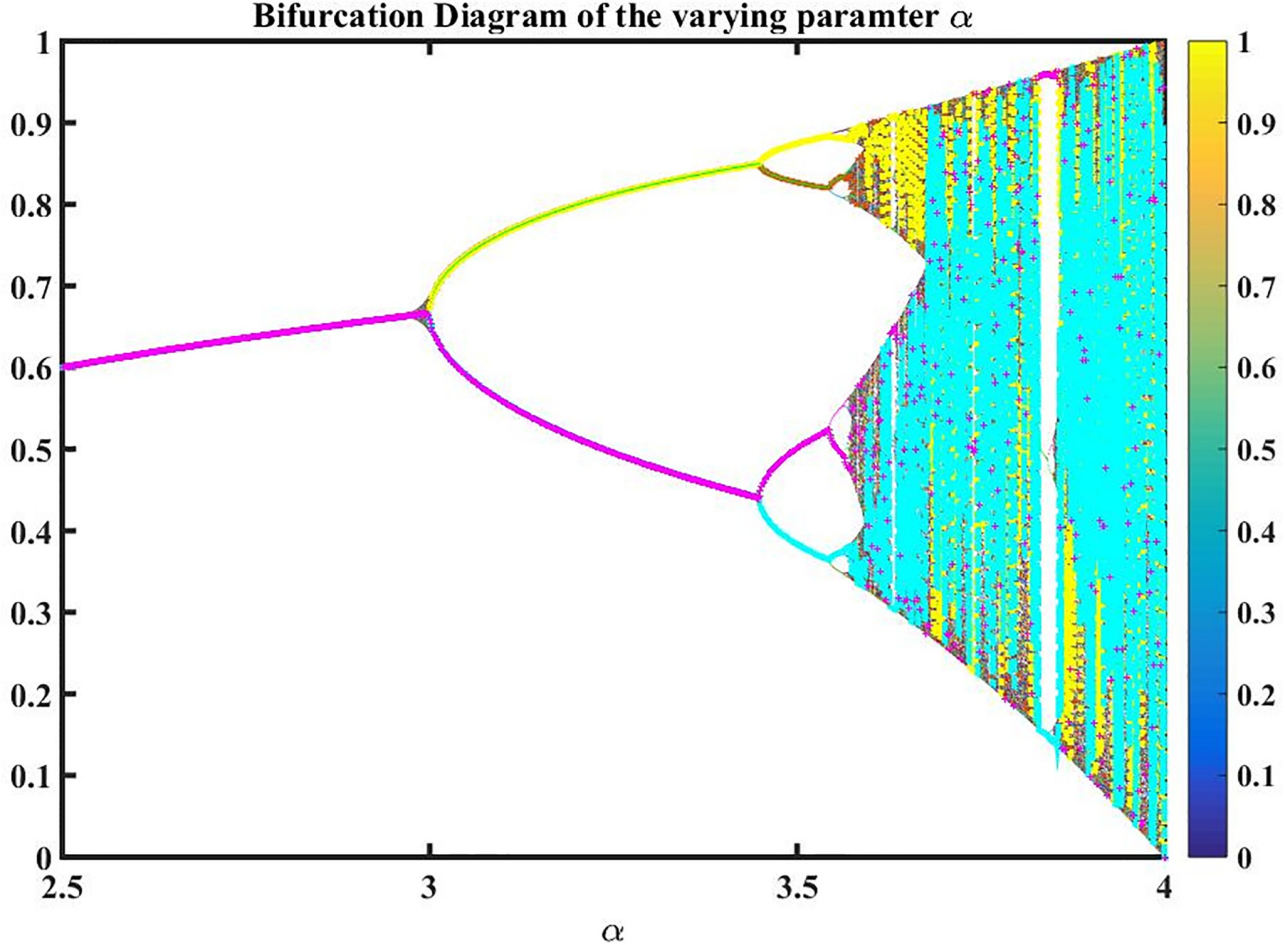
Bifurcation diagram of varying the parameter value of *α* as shown in Table 1.

An effective method for comprehending the complex behavior of dynamical systems under parameter variation is through numerical simulation of bifurcation diagrams. Researchers can examine the system’s response to changes in the regulating parameter and spot important patterns and transitions as shown in Fig 1. This approach is frequently used to understand the behavior of complex systems in a variety of disciplines, including physics, biology, economics, and engineering.

**Table 1:** Represent the numerical data of varying *α* parameter along bifurcation and Lyapunov exponent.

TIME	*a*_1_	*a*_2_	*a*_3_	Lyapunov exponents	Bifurcation analysis
10.00	3.5	5.12	-1.9	1.4	1.78
20.00	3.6	5.94	-1.5	1.44	2
30.00	3.9	5.44	-1.7	1.5	2.1
40.00	3.9	5.74	-1.8	1.56	3.2
50.00	4.10	5.24	-1.8	1.6	2.2
60.00	4.06	5.44	-1.9	1.76	2.26
70.00	4.1	5.42	-2.9	1.8	2.43
80.00	5.1	5.14	-2.9	1.9	2.5

### Perturbed dynamical system

A dynamical system is said to be perturbed when external disturbances or changes in its properties occur. When perturbations are present, the behavior of the system can be dramatically altered, deviating from that of the corresponding nonperturbed system. Studying how these perturbations affect the dynamics of a system is part of the analysis of perturbed dynamical systems [34]. The existence of chaotic behavior can have significant consequences when studying a perturbed dynamical system. The term “chaotic behavior” describes the intricate, erratic, and delicate quality of a system’s trajectories, which frequently result from the interaction of nonlinear dynamics and outside shocks. Here is an example of how chaotic behavior might impact how a disrupted dynamical system is analyzed. It is crucial to take into account the unique properties of the perturbations and the system’s reaction to them when researching a chaotic dynamical system. To analyze and forecast the system’s behavior under perturbations, sophisticated mathematical techniques like nonlinear dynamics and chaos theory are frequently used.

A dynamical system’s Lyapunov exponents are essential in assessing whether chaos exists there. They offer a numerical assessment of the system’s sensitivity to the starting conditions and can show whether the system behaves chaotically. The system shows sensitivity to initial conditions if at least one Lyapunov exponent is positive. An essential trait of chaotic behavior is this sensitivity. Positive Lyapunov exponents reveal the existence of exponential trajectory divergence and the sensitive dependency on beginning conditions, indicating chaos and the system’s unpredictable nature. The perturbed dynamical system for Eq (13), as follows:

$$\left\{ \begin{array}{c} \frac{dP}{d\Psi }=U\\ \frac{dU}{d\Psi }=-2f_{1}\left(P^{3}\right)-2f_{2}P+\varepsilon \;\text{cos}\;\left(\alpha \psi \right)=V\\ \frac{dE}{d\Psi }=\alpha , \end{array}\right.$$ (56)

where frequency *ψ* and amplitude *ε*. In this investigation, the impact of *ψ* and *ε* on the governing model will be examined. We have demonstrated the chaotic behavior of system Eq (56) using a range of *ε* and *ψ* values as well as other suitable values of a parameter, as shown in Figs 17 to 20.

#### Lyapunov exponents

**Fig 2:**
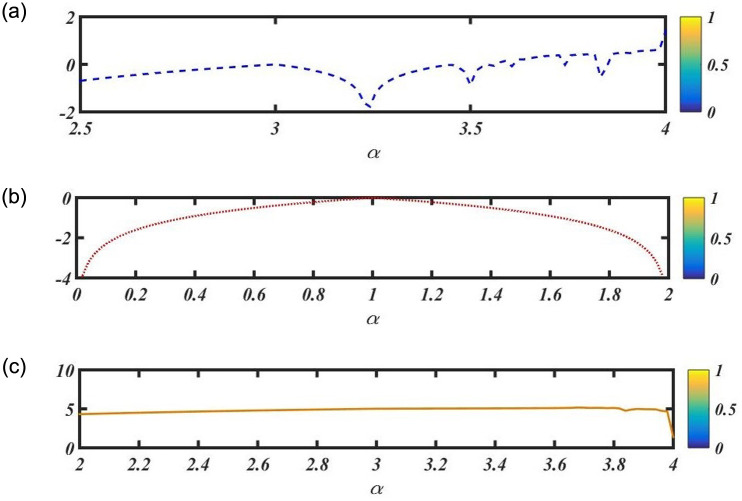
Physical depiction of Eq (56), Fig. (*a*_1_), when values of parameter is *α* = 1.3, Fig. (*a*_2_), when values of parameter is *α* = 2.3 and Fig. (*a*_3_), when values of parameter is *α* = 3.1, as shown in Table 1.

Lyapunov exponent numerical simulation is a potent technique for comprehending the behavior of complicated dynamical systems. Researchers can use it to look into the existence of chaos, pinpoint areas of stability, and analyze how governing parameters affect the dynamics of the system. However, the results must be interpreted with care and a thorough knowledge of the underlying dynamics of Eq (56). Lyapunov exponents frequently rely on the characteristics of the system. To investigate the connection between Lyapunov exponents and regulating variables, bifurcation analysis can be used. The following Fig 2, represents the varying parameter *α* of Lyapunov exponents.

## Results and discussions

It is crucial to assess the improvements and contributions made by the new research by comparing it to earlier work. We can determine the novelty, significance and possible influence by analyzing and contrasting the innovative work with the previous work in the literature. A thorough study, the drawbacks of earlier methods and any prospective advancements or innovations brought about by the innovative work are all made possible by this comparison. The comparison serves to prove the importance and relevance of the new work within the larger scientific community by highlighting the advancements and filling in the gaps in earlier studies. Kocak et al., find the traveling solutions for the CKOM by using the technique of modified exp-function. In this research, we obtained novel solitons by using the unified technique. Also, obtained the phase portrait analysis for a perturbed and nonperturbed dynamical system. By employing the UT to FCKOM, get the novel soliton solutions which are hyperbolic, rational and trigonometric. The innovative soliton solutions to the FCKOM that were discovered utilizing the UT may provide important new understandings into a number of physical processes. Understanding wave dynamics, signal propagation, and other nonlinear systems frequently rely heavily on soliton solutions. The novel approaches could lead to the discovery of novel patterns and behaviors in complex systems.

By effectively, precisely and adaptably resolving the FCKOM complexity, the UT represents a substantial achievement in the discipline. It is a strong and useful tool for researchers looking at complicated systems including fractional calculus and coupled dynamics because of its capacity to find new soliton solutions, provide analytical insights and handle a wide range of nonlinear equations. Eqs (18)–(25) and (36)–(43) are hyperbolic solutions. Eqs (26)–(33) and (44)–(51) are trigonometric solutions. Eqs (34), (35), (52) and (53) are rational solutions.

A specific kind of solution that appears in a number of nonlinear wave equations and demonstrates both soliton and hyperbolic properties is called a hyperbolic solution. Shockwave propagation, conduction of heat, and dispersion problems have solutions that include hyperbolic functions. They offer crucial insights into how these dynamic processes behave, such as the distribution of temperature along a rod. Trigonometric functions are essential for understanding system behavior and designing controls for mechanical oscillations, alternating currents, and voltages. Filtering and frequency analysis are examples of rational functions used in signal processing. They give engineers the ability to work with signals and retrieve crucial information. Applications for these solutions can be found in the study of nonlinear dynamics as well as in the domains of optics, fluid dynamics, and plasma physics. Trigonometric solutions offer an effective foundation for delving into and comprehending a range of mathematical and physical issues. They are a vital instrument in mathematics, physics, engineering, and other disciplines due to their periodic character, association with simple harmonic motion, and applications in numerous fields. For solving equations, modeling relationships, and analyzing systems, rational solutions provide a flexible framework. Their depiction as polynomial ratios allows for a deep understanding of mathematical processes and makes it easier to use them practically in a variety of disciplines, including engineering, physics, statistics and data analysis.

### Physical interpretation

**Fig 3:**
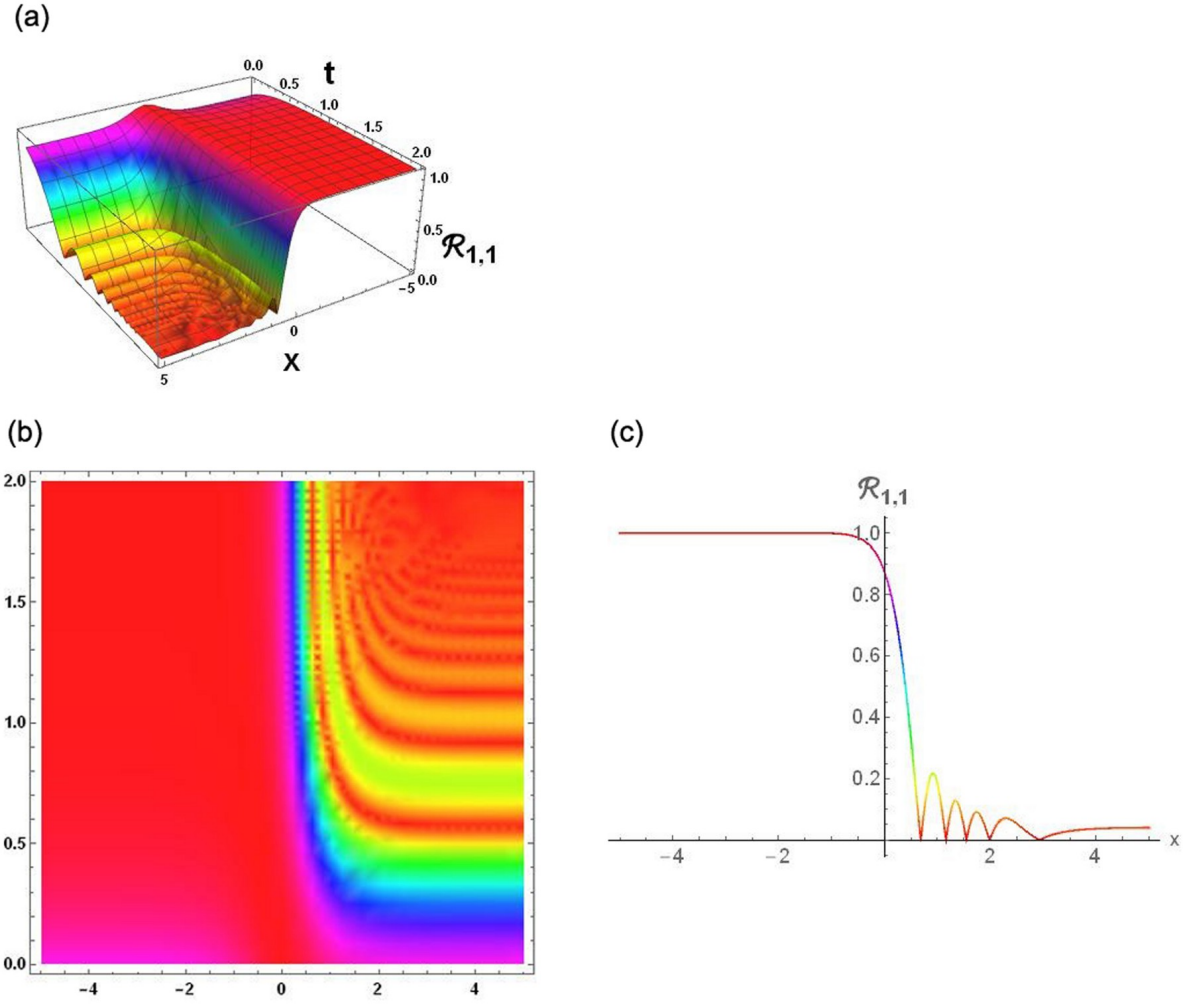
Physical depiction of Eq (18), of hyperbolic solutions under the suitable values of parameter are *σ* = −0.1, *L* = 2.1, *h* = 0.7, *l* = 1.6, *δ* = 0.2, *γ* = 0.6, *τ* = 1.3, *s* = 0.55 & *q* = 1.4.

**Fig 4:**
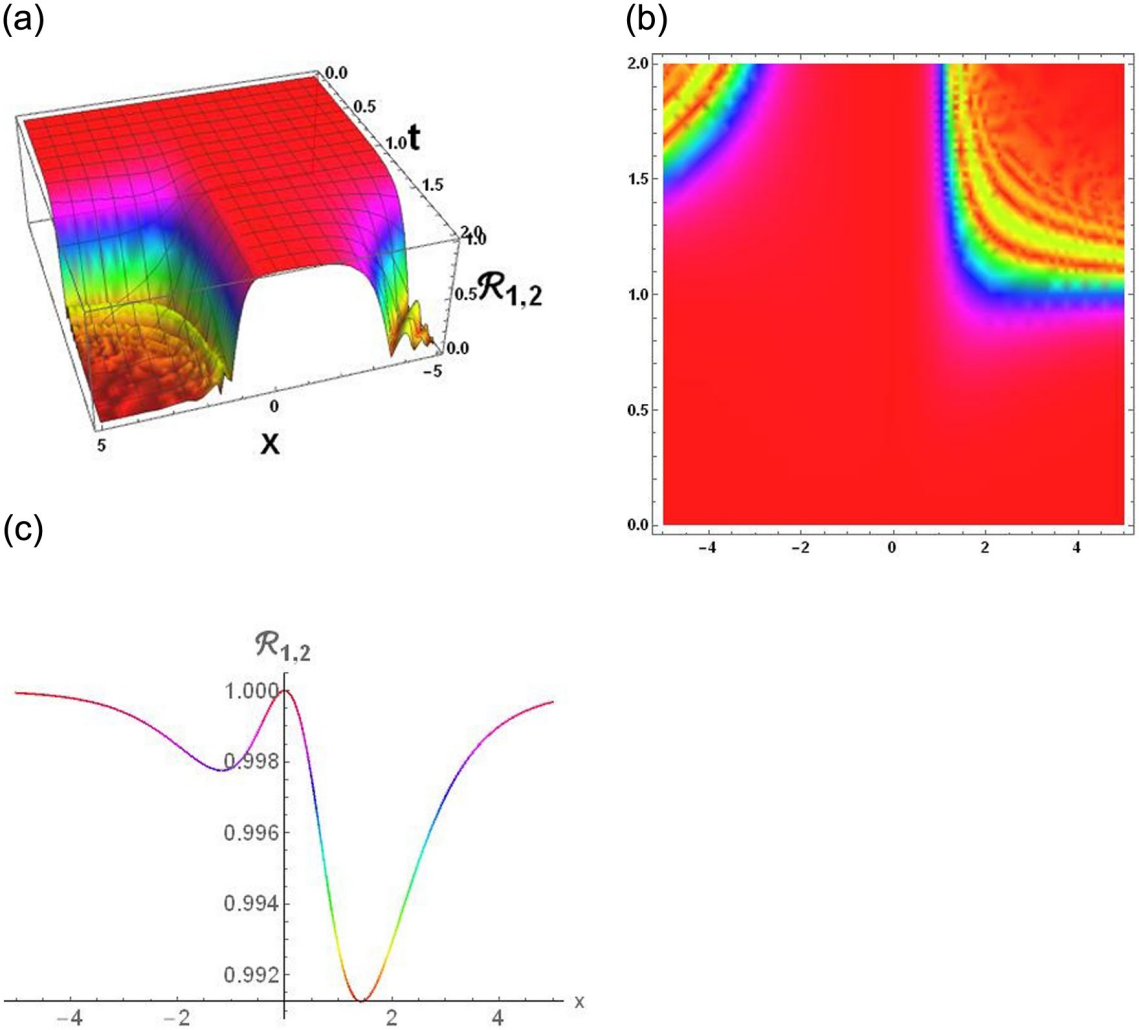
Physical depiction of Eq (20), of hyperbolic solutions under the suitable values of parameter are *σ* = −0.4, *L* = 2.1, *h* = 0.7, *l* = 1.6, *δ* = 0.2, *γ* = 0.6, *τ* = 1.3, *s* = 0.55 & *q* = 1.4.

**Fig 5:**
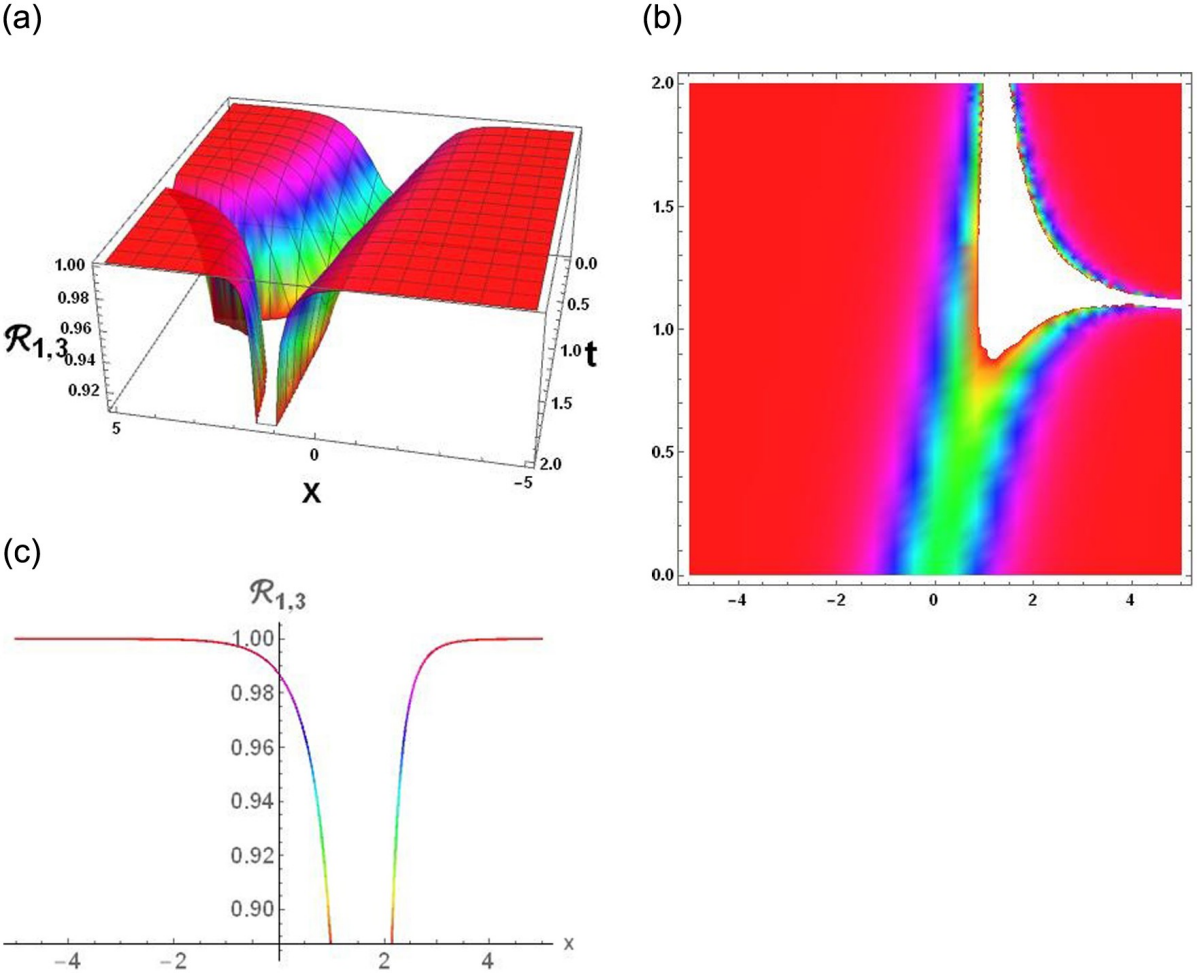
Physical depiction of Eq (22), of hyperbolic solutions under the suitable values of parameter are *σ* = −0.8, *L* = 2.1, *h* = 0.7, *l* = 1.6, *δ* = 0.2, *γ* = 0.6, *τ* = 1.3, *s* = 0.55 & *q* = 1.4.

**Fig 6:**
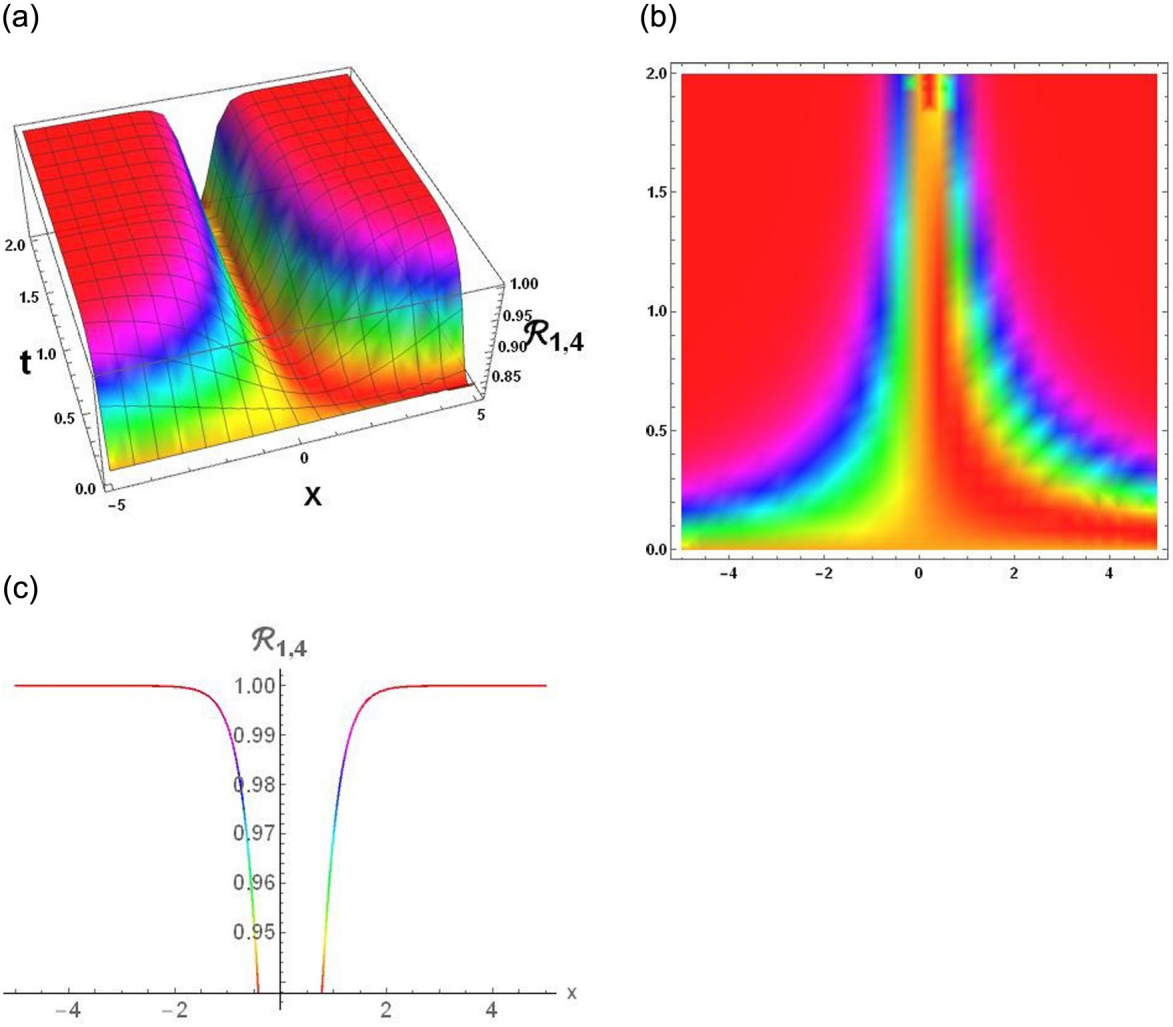
Physical depiction of Eq (24), of periodic solutions under the suitable values of parameter are *σ* = −0.34, *L* = 2.1, *h* = 0.7, *l* = 1.6, *δ* = 0.2, *γ* = 0.6, *τ* = 1.3, *s* = 0.55 & *q* = 1.4.

**Fig 7:**
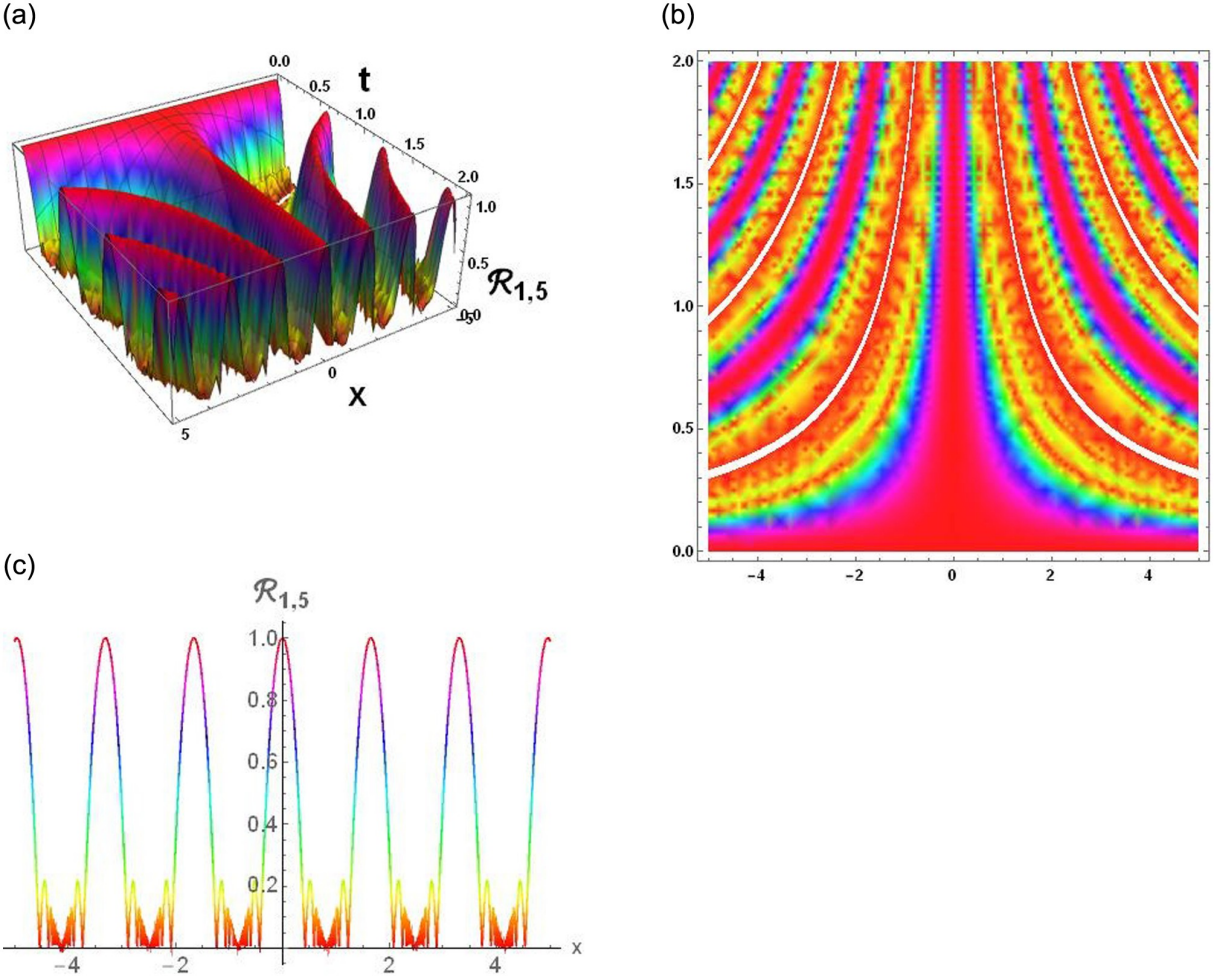
Physical depiction of Eq (26), of hyperbolic solutions under the suitable values of parameter are *σ* = 0.1, *L* = 2.1, *h* = 0.7, *l* = 1.6, *δ* = 0.2, *γ* = 0.6, *τ* = 1.3, *s* = 0.55 & *q* = 1.4.

**Fig 8:**
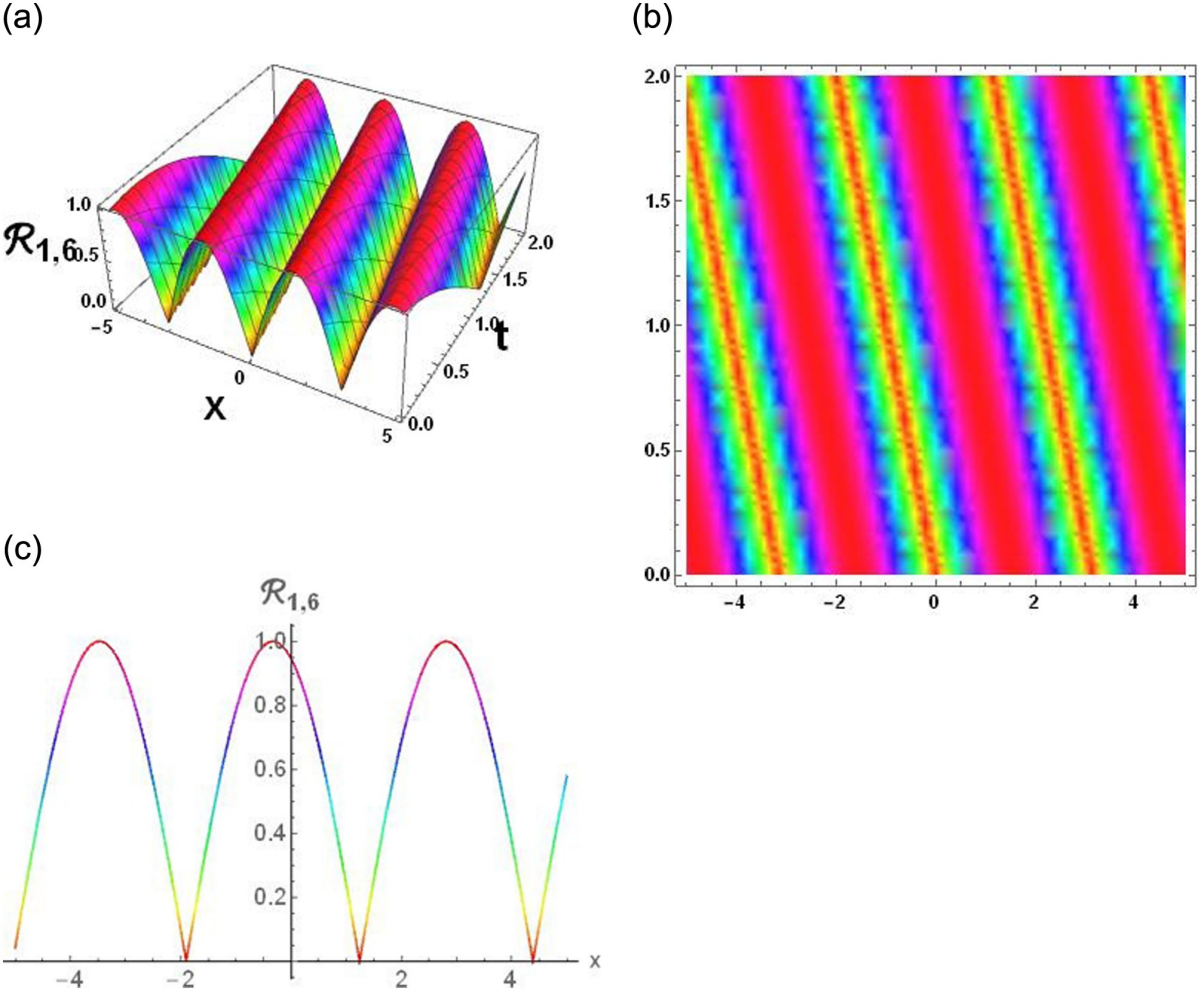
Physical depiction of Eq (28), of periodic solutions under the suitable values of parameter are *σ* = 0.4, *L* = 2.1, *h* = 0.7, *l* = 1.6, *δ* = 0.2, *γ* = 0.6, *τ* = 1.3, *s* = 0.55 & *q* = 1.4.

**Fig 9:**
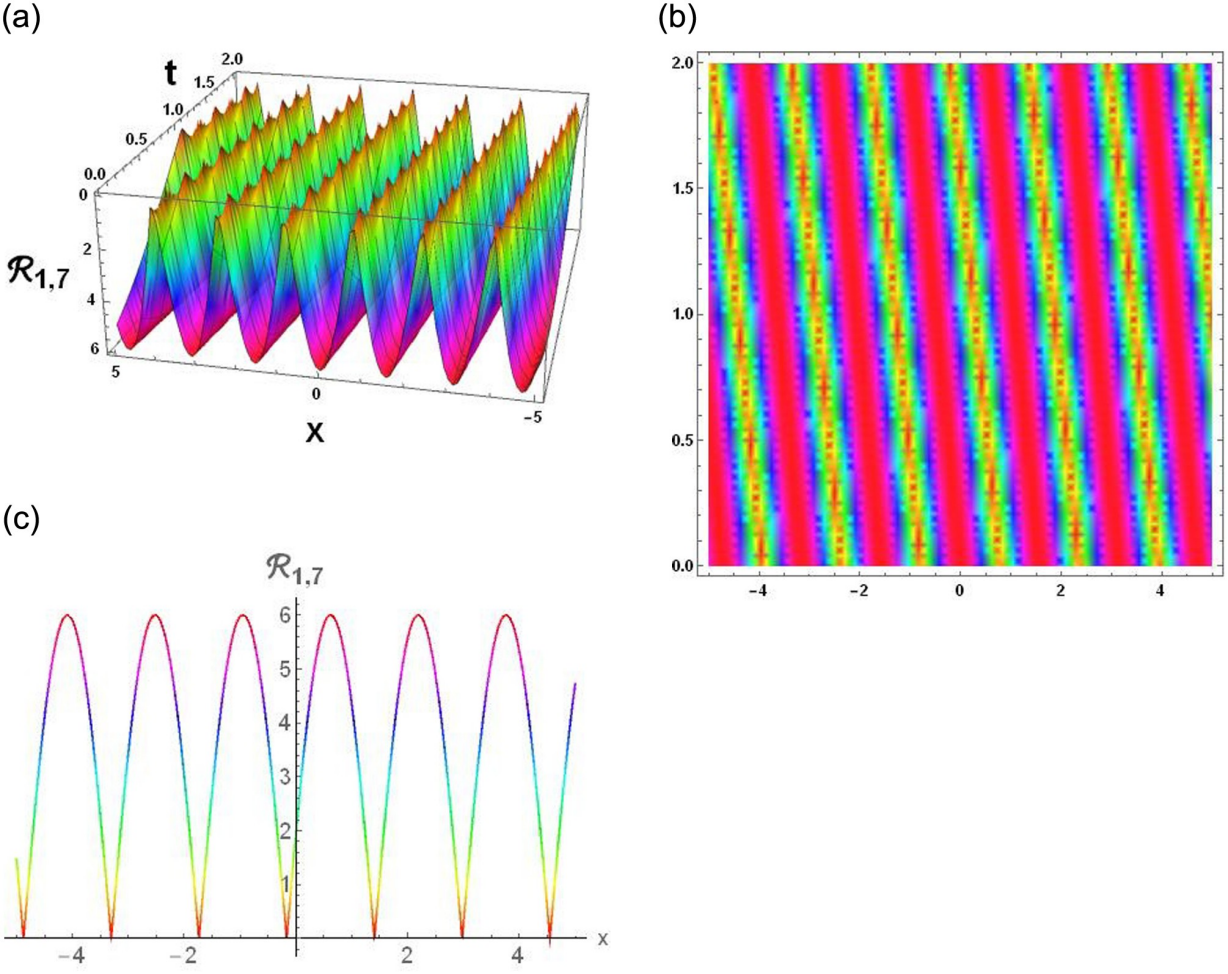
Physical depiction of Eq (30), of trigonometric solutions under the suitable values of parameter are *σ* = 0.99, *L* = 2.1, *h* = 0.7, *l* = 1.6, *δ* = 0.2, *γ* = 0.6, *τ* = 1.3, *s* = 0.55 & *q* = 1.4.

**Fig 10:**
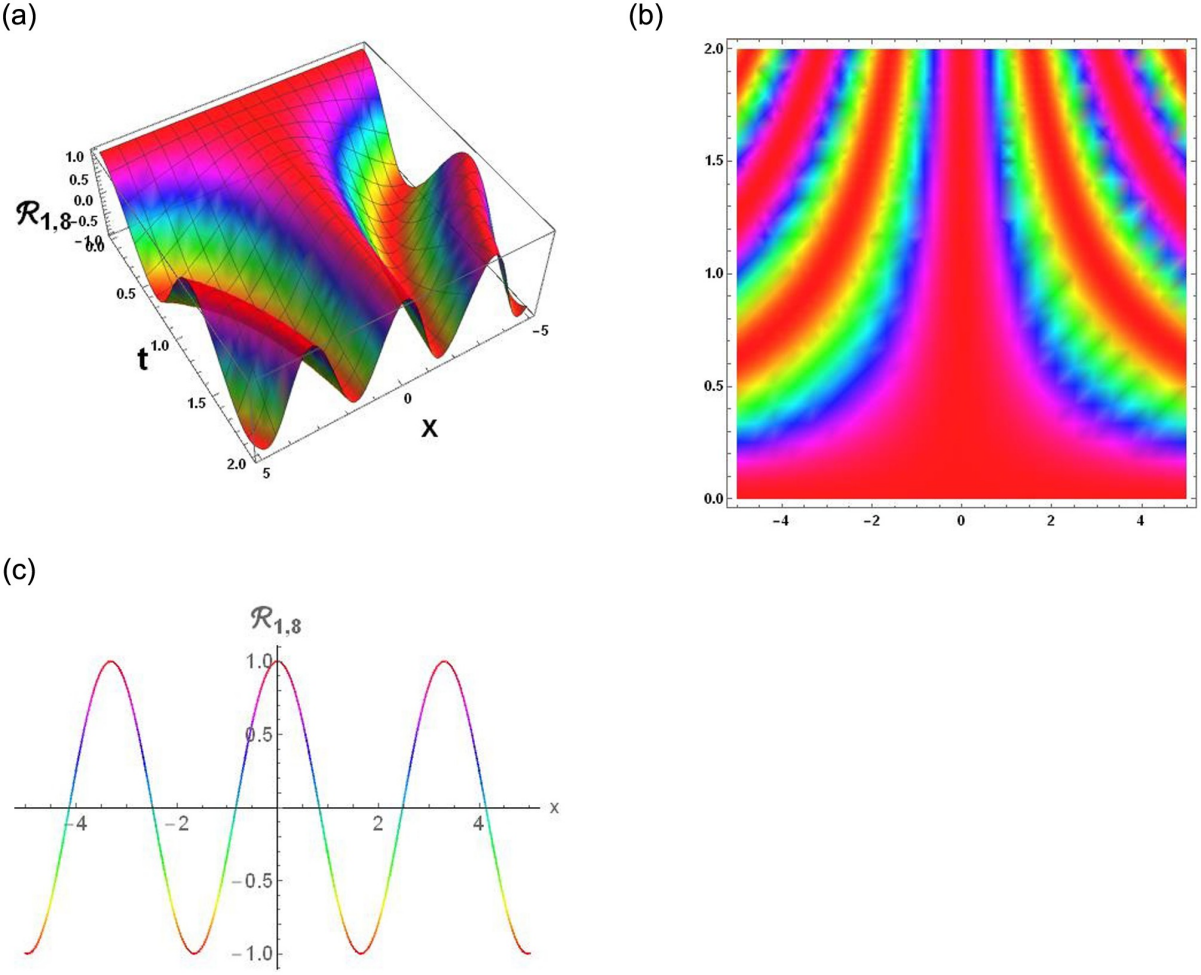
Physical depiction of Eq (32), of periodic solitary wave solutions under the suitable values of parameter are *σ* = 0.44, *L* = 2.1, *h* = 0.7, *l* = 1.6, *δ* = 0.2, *γ* = 0.6, *τ* = 1.3, *s* = 0.55 & *q* = 1.4.

**Fig 11:**
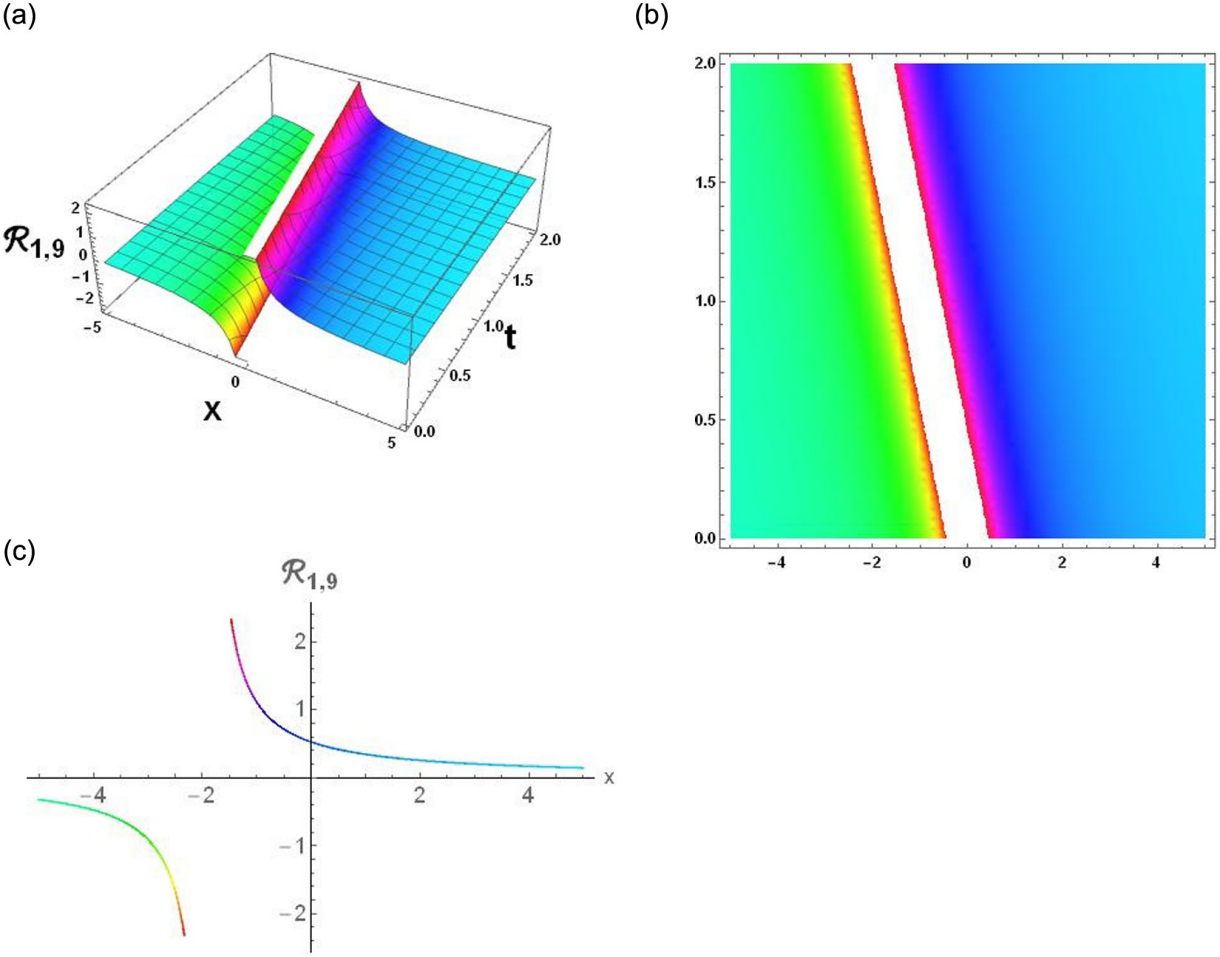
Physical depiction of Eq (34), of rational solutions under the suitable values of parameter are *σ* = 0, *L* = 2.1, *h* = 0.7, *l* = 1.6, *δ* = 0.2, *γ* = 0.6, *τ* = 1.3, *s* = 0.55 & *q* = 1.4.

**Fig 12:**
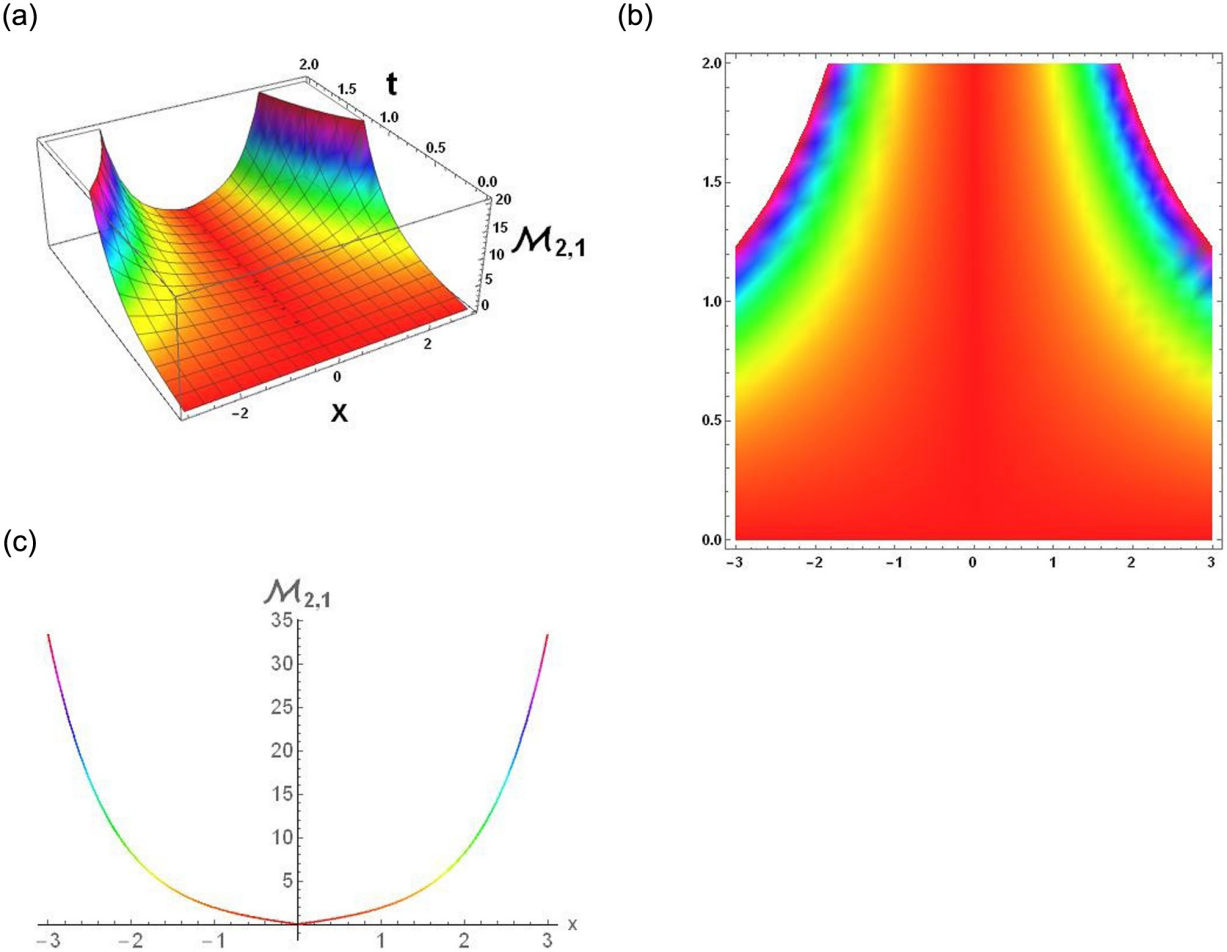
Physical depiction of Eq (37), of U-shaped singular soliton under the suitable values of parameter are *σ* = −1.3, *L* = 2.1, *h* = 0.7, *l* = 1.6, *δ* = 0.2, *γ* = 0.6, *τ* = 1.3, *s* = 0.55 & *q* = 1.4.

**Fig 13:**
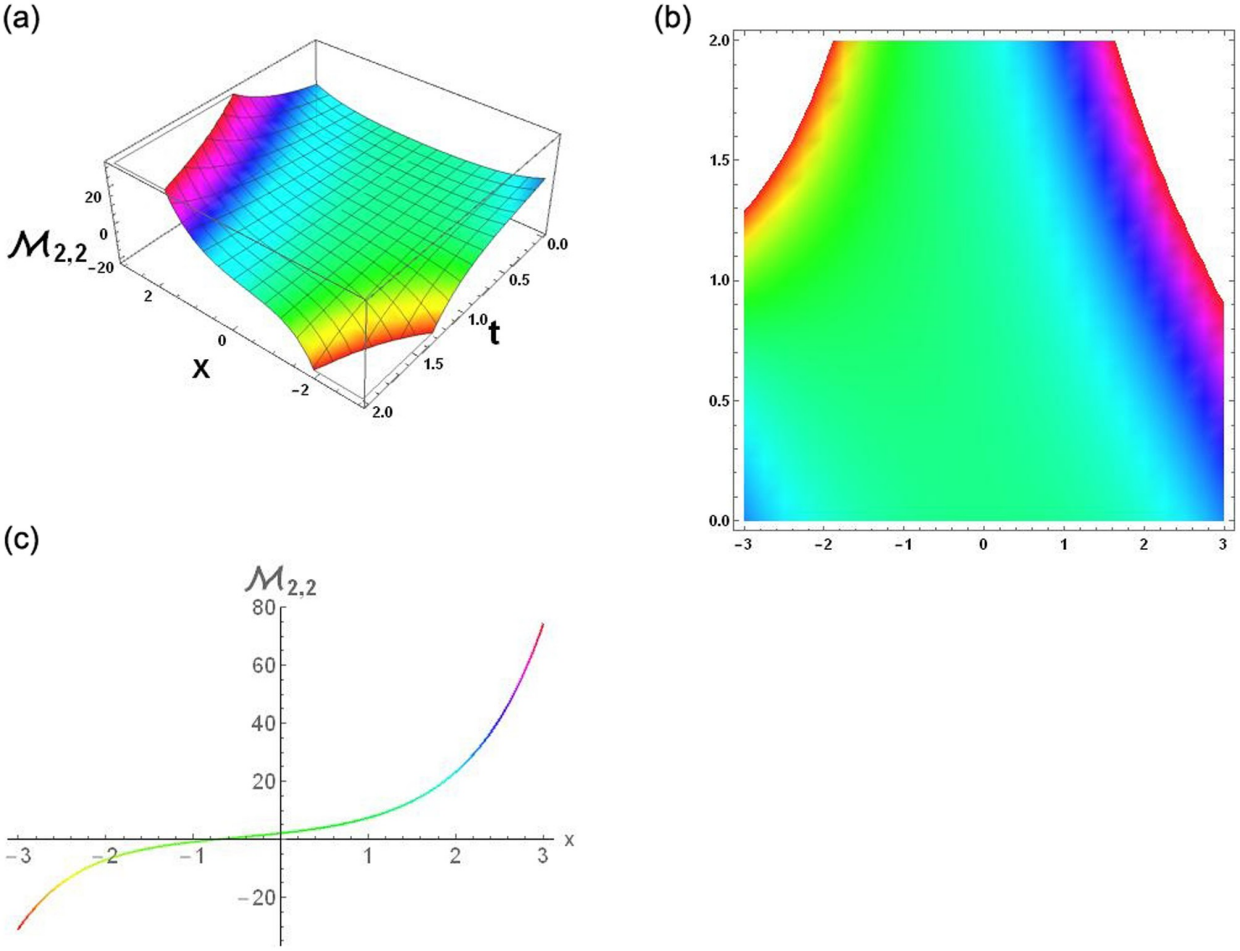
Physical depiction of Eq (39), of hyperbolic solutions under the suitable values of parameter are *σ* = −0.5, *L* = 2.1, *h* = 0.7, *l* = 1.6, *δ* = 0.2, *γ* = 0.6, *τ* = 1.3, *s* = 0.55 & *q* = 1.4.

**Fig 14:**
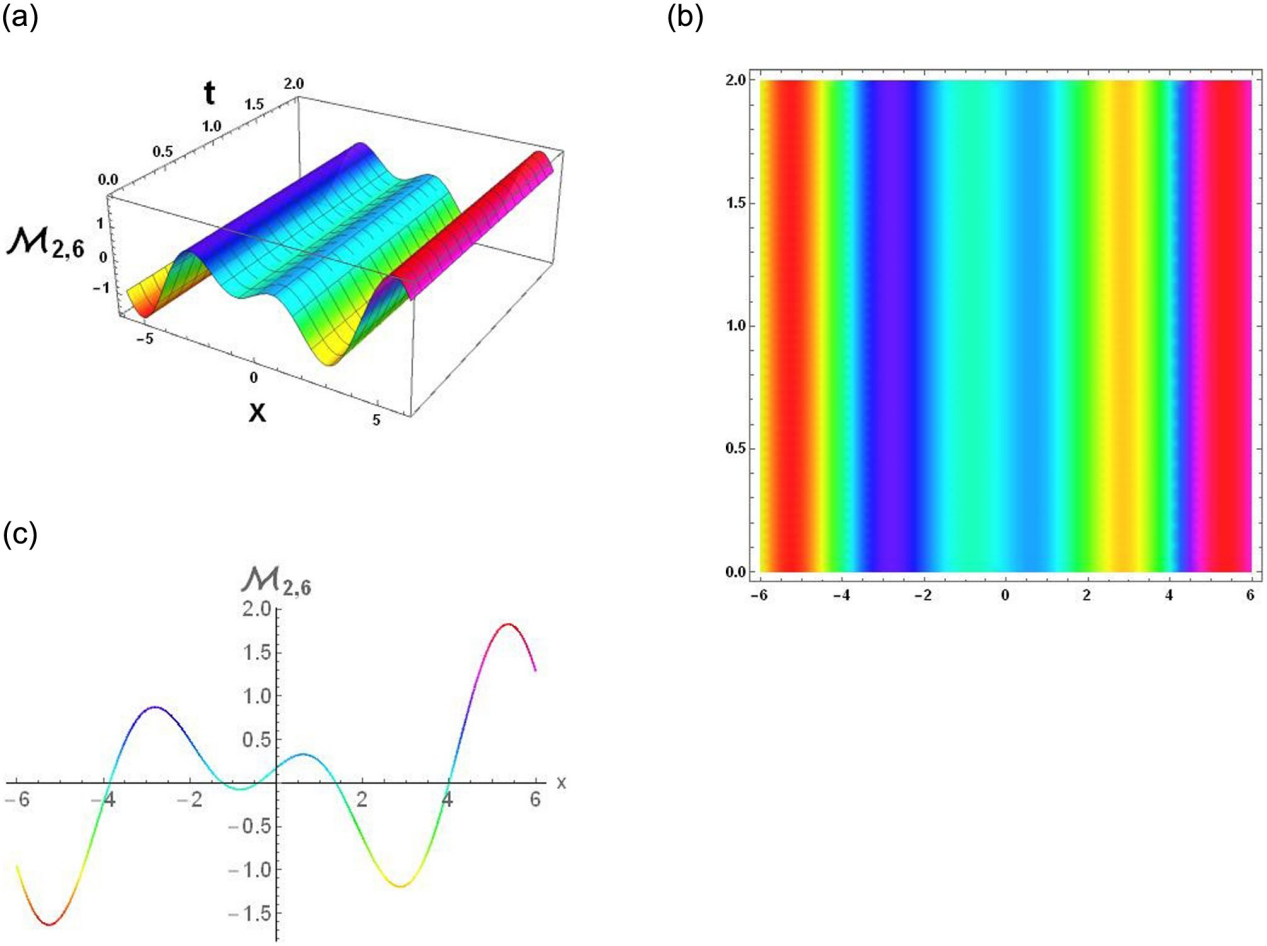
Physical depiction of Eq (47), of trigonometric solutions under the suitable values of parameter are *σ* = 1.4, *L* = 2.1, *h* = 0.7, *l* = 1.6, *δ* = 0.2, *γ* = 0.6, *τ* = 1.3, *s* = 0.55 & *q* = 1.4.

The results that were obtained are covered in this section, along with the variety of solutions that were found for the model defined by Eq (2), hyperbolic, trigonometric and rational solutions are among the solutions that have been found. Numerous scientific and engineering disciplines use hyperbolic, trigonometric, and rational solutions. For example, physics, electrical engineering, control theory, wave phenomena, electrical engineering, mechanical engineering, logical solutions, control systems, signal processing, and fluid dynamics. Then, using the proper parameter values, we yield graphs in the 2D, density plot and 3D formats to visually illustrate these solutions. Figs 3–6, represents the hyperbolic solutions for $R_{1,1},\;R_{1,2},\;R_{1,3}\;\&\;R_{1,4}$. Similarly, $M_{1,1},\;M_{1,2},\;M_{1,3}\;\&\;M_{1,4}$, are also the solution of hyperbolic. Figs 7–10, represents the trigonometric solutions for $R_{1,5},\;R_{1,6},\;R_{1,7}\;\&\;R_{1,8}$. Similarly, $M_{1,5},\;M_{1,6},\;M_{1,7}\;\&\;M_{1,8}$, are also the solution of trigonometric. Fig 11, represent the plane wave solution for $R_{1,9}$. Similarly, $M_{1,9}$, $R_{2,9}\;\&\;M_{2,9}$ are also the solution of plane wave. Figs 12 & 13, represents the hyperbolic solutions for $M_{2,1}\;\&\;M_{2,2}$. Similarly, $M_{2,3},\;M_{2,4},\;R_{2,1},\;R_{2,2}\;R_{2,3}\;\&\;M_{2,4}$, are also the solution of hyperbolic. Fig 14, represent the trigonometric solutions for $M_{2,6}$. Similarly, $M_{2,5},\;M_{2,7},\;M_{2,8},\;R_{2,5},\;R_{2,6}\;R_{2,7}\;\&\;R_{2,8}$, are also the solution of trigonometric.

Figs 15–18, represent the phase analysis for an unperturbed dynamical system. Figs 19–22, represent the phase analysis for a perturbed dynamical system. Fig 23, represents the Lyapunov exponent for the existence of chaos dynamical system.

**Fig 15:**
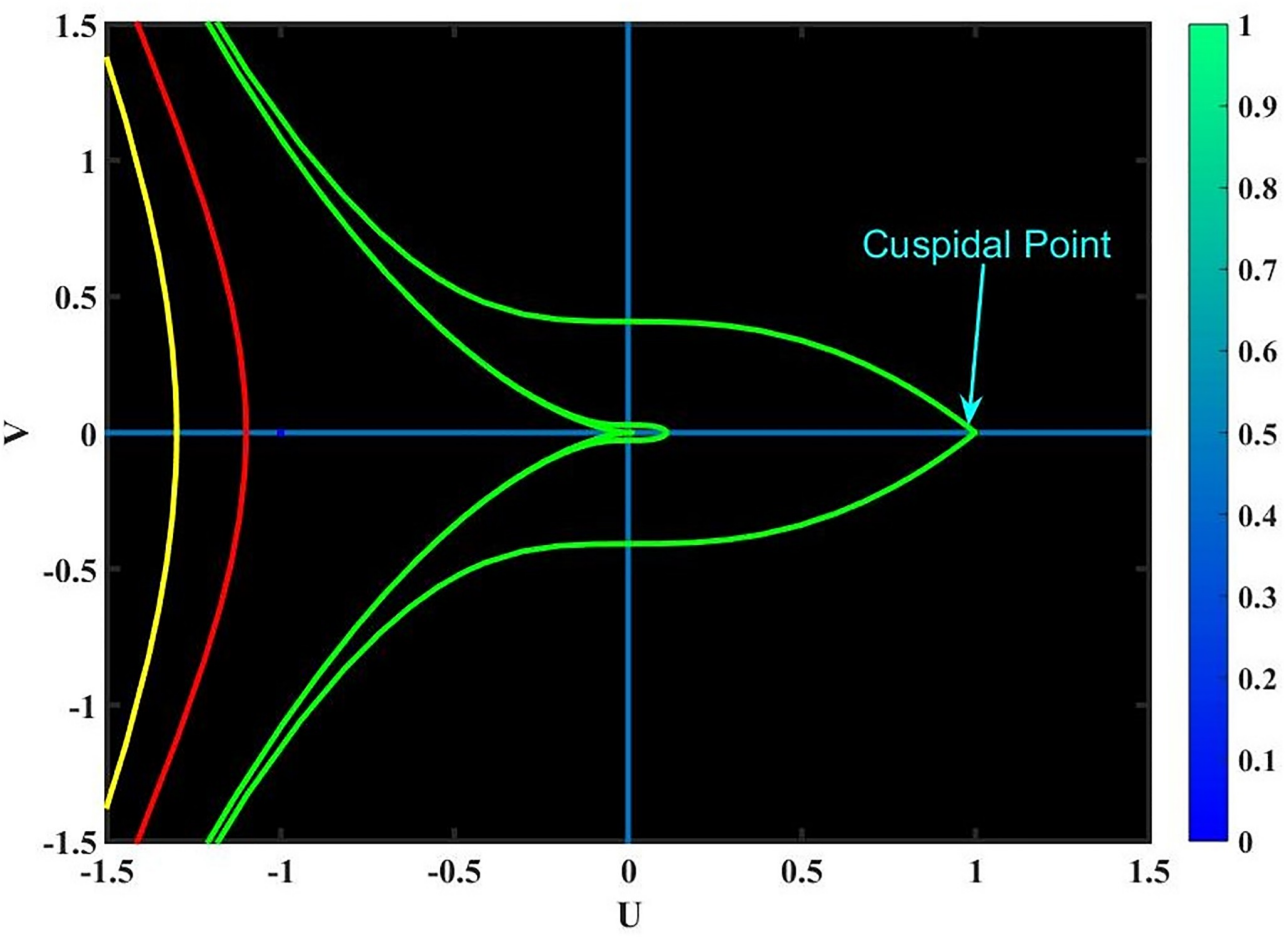
Physical depiction for (54) of a case-(4.1), which shows the behavior of cuspidal points under suitable parametric conditions *s* = −0.3, *q* = −1.1 & *L* = 1.2.

**Fig 16:**
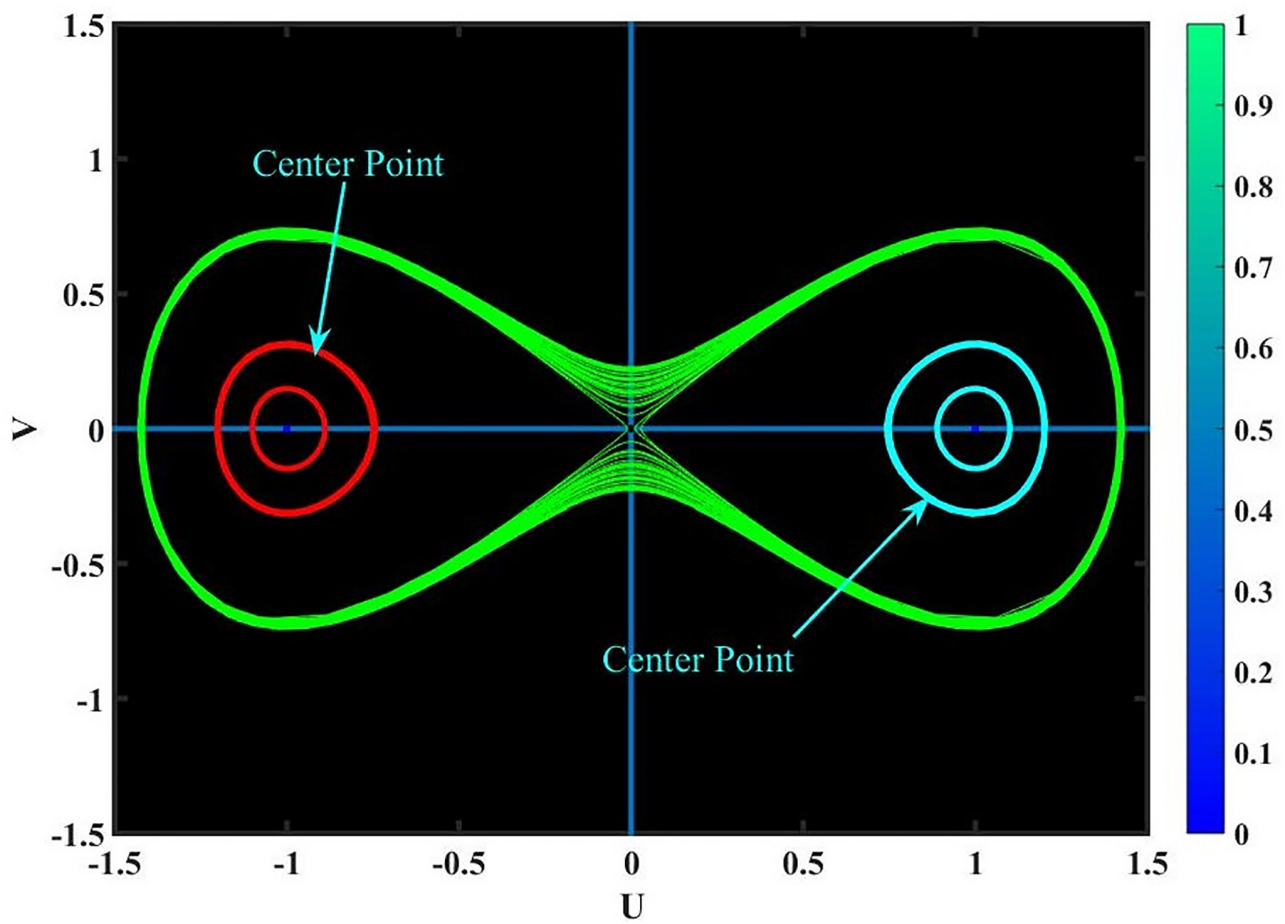
Physical depiction for (54) of case-(4.2), which shows the behavior of center points under suitable parametric conditions *s* = −0.23, *q* = 1.45 & *L* = 1.4.

**Fig 17:**
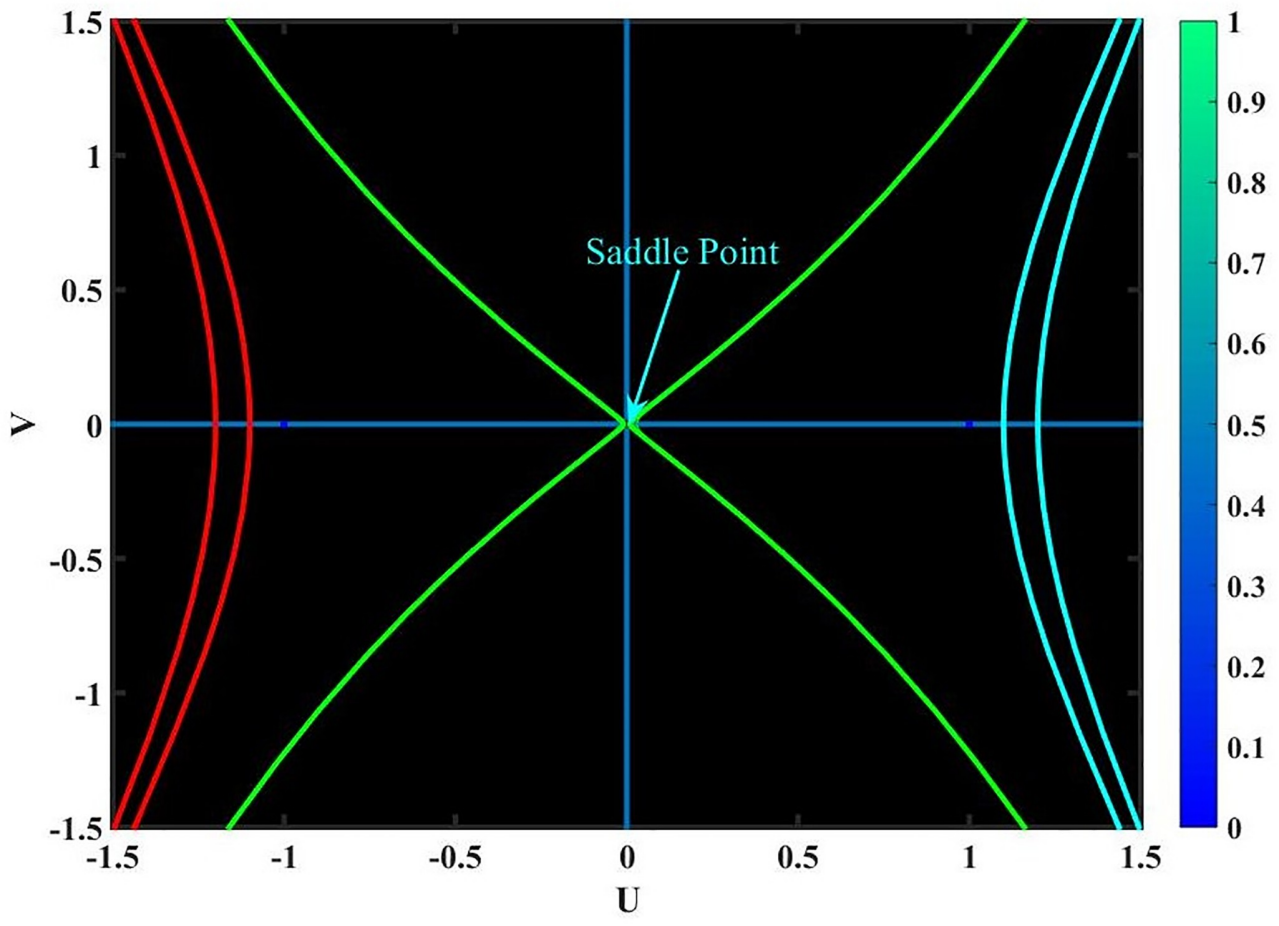
Physical depiction for (54) of case-(4.3), which shows the behavior of saddle points under suitable parametric conditions *s* = 0.4, *q* = −1.5 & *L* = 1.24.

**Fig 18:**
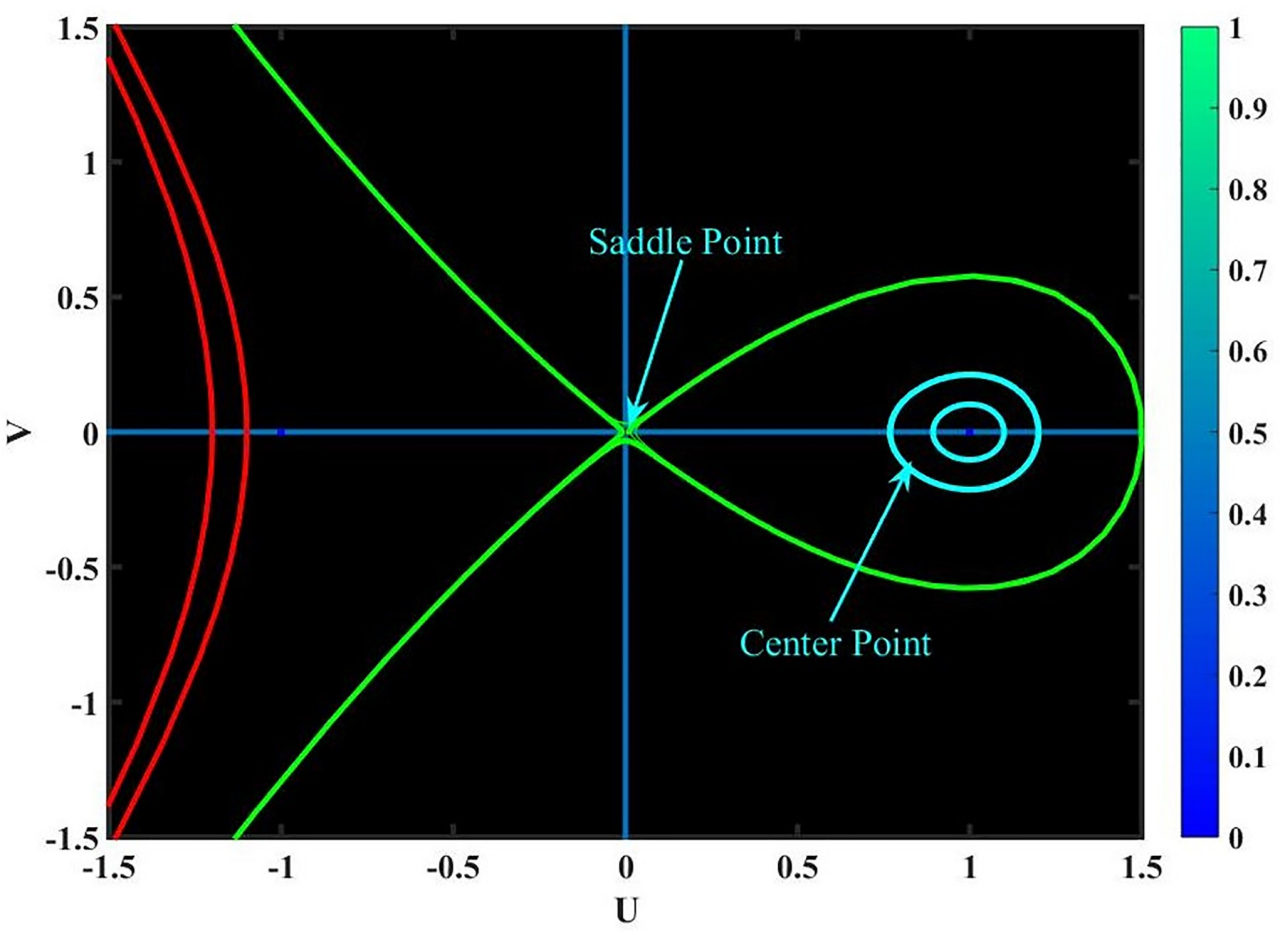
Physical depiction for (54) of case-(4.4), which shows the behavior of saddle and center points under suitable parametric conditions *s* = 1.4, *q* = 2.5 & *L* = 1.5.

**Fig 19:**
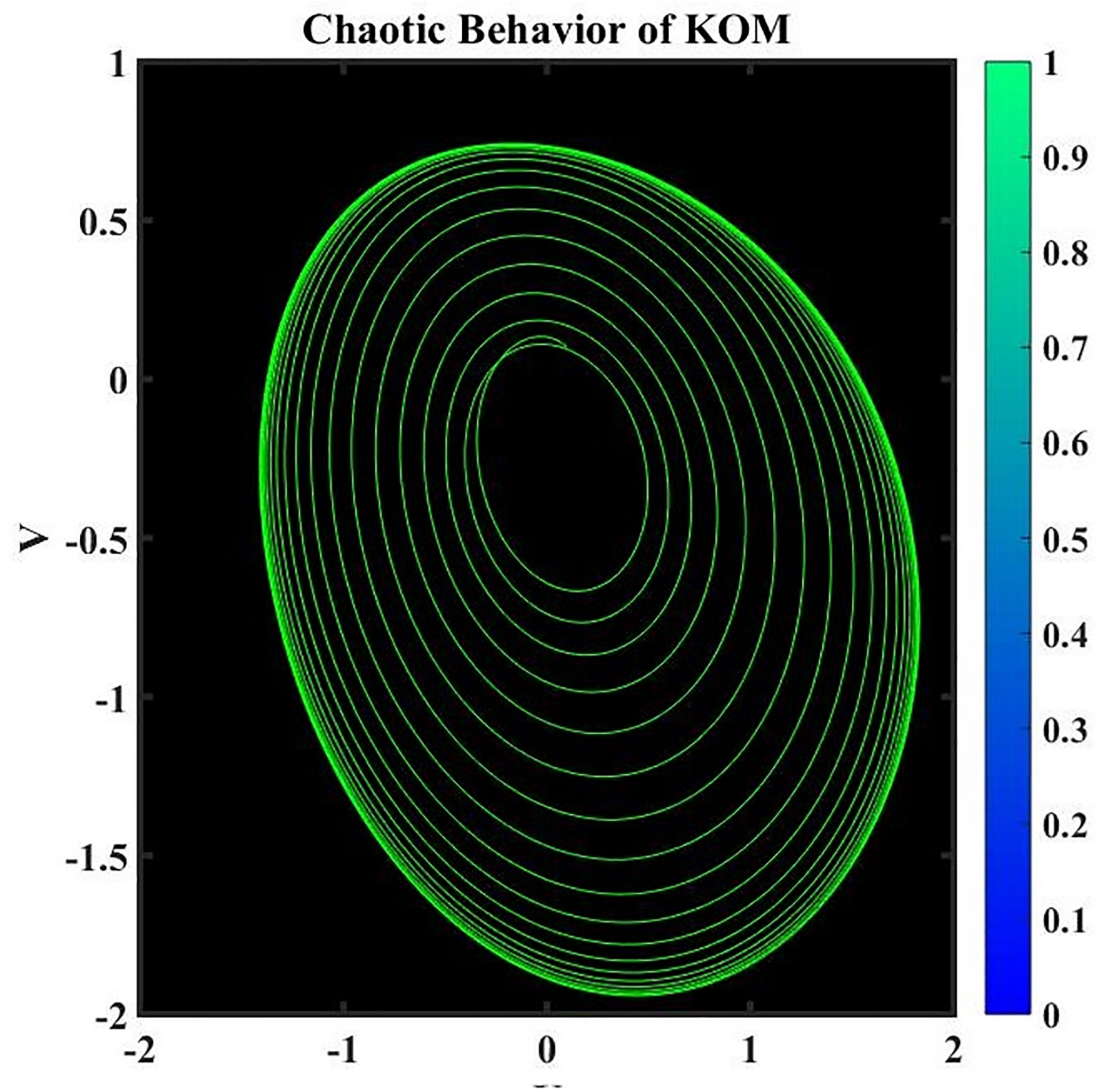
Physical depiction for chaotic behavior of (56), when *ε* = 1.3 & *ψ* = 0.1, and the suitable parametric values are *s* = 0.14, *q* = −1.35 & *L* = 1.12.

**Fig 20:**
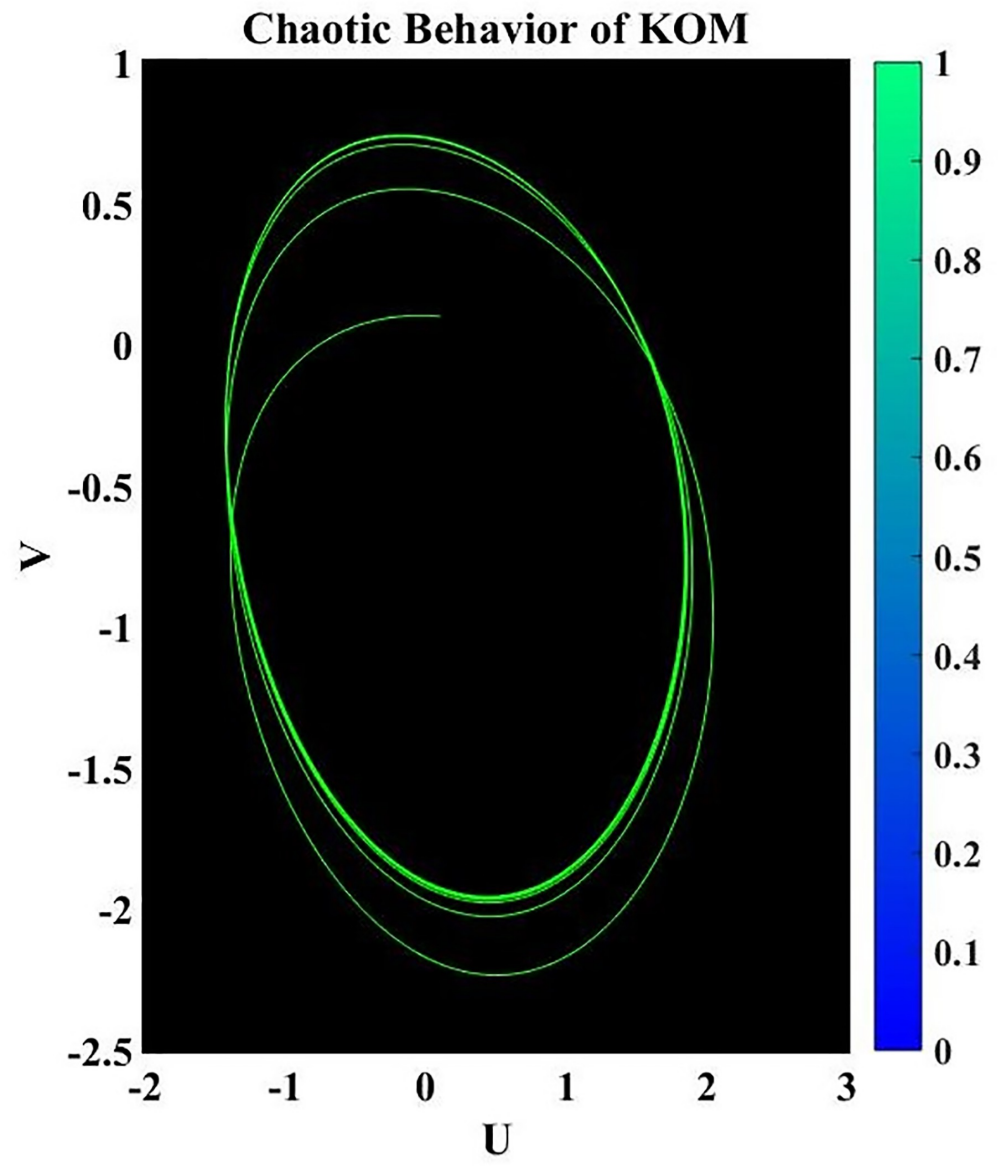
Physical depiction for chaotic behavior of (56), when *ε* = 0.2 & *ψ* = 0.01, and the suitable parametric values are *s* = 1.13, *q* = −2.32 & *L* = 3.12.

**Fig 21:**
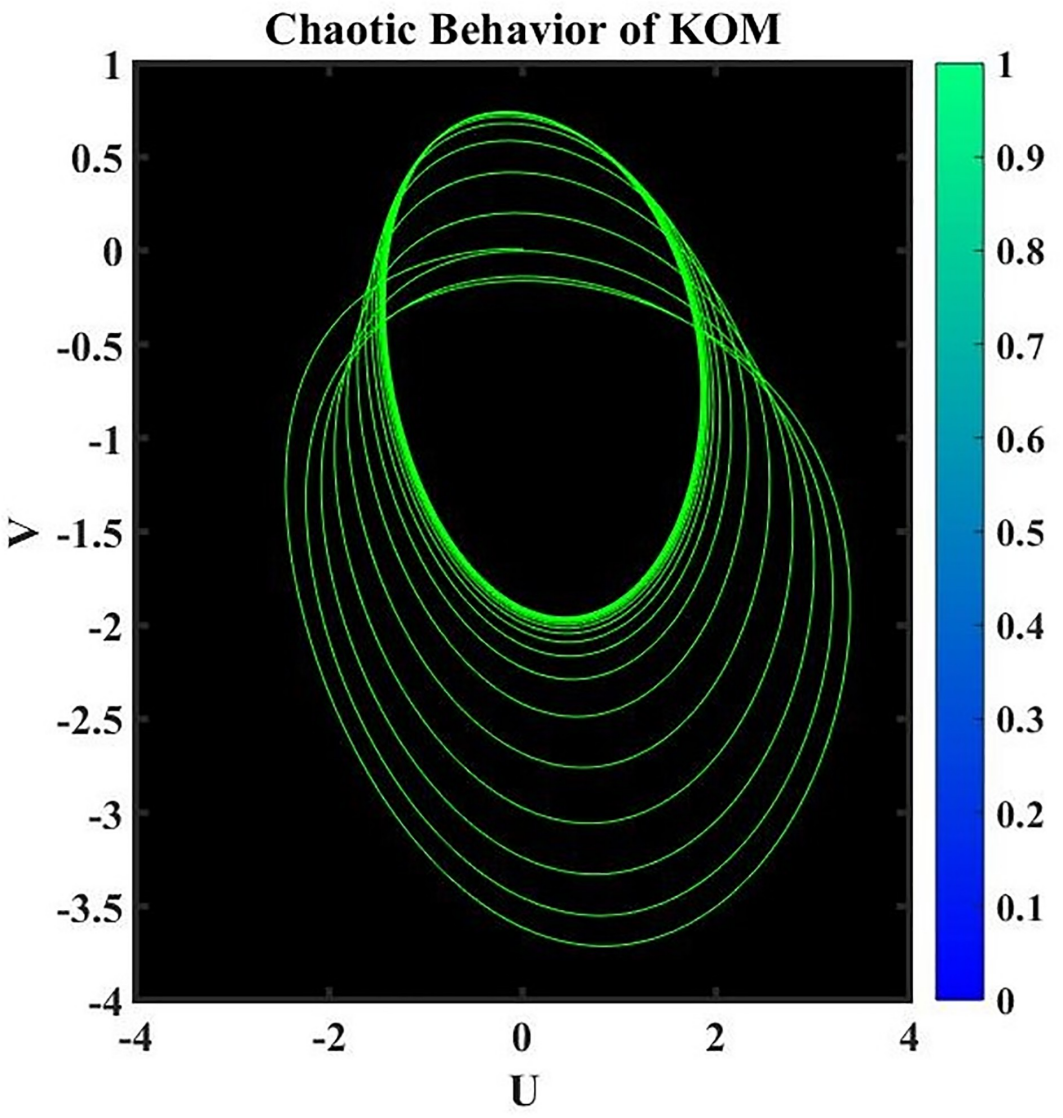
Physical depiction for chaotic behavior of (56), when *ε* = 1.4 & *ψ* = 2.1, and the suitable parametric values are *s* = 4.14, *q* = −1.5 & *L* = 0.12.

**Fig 22:**
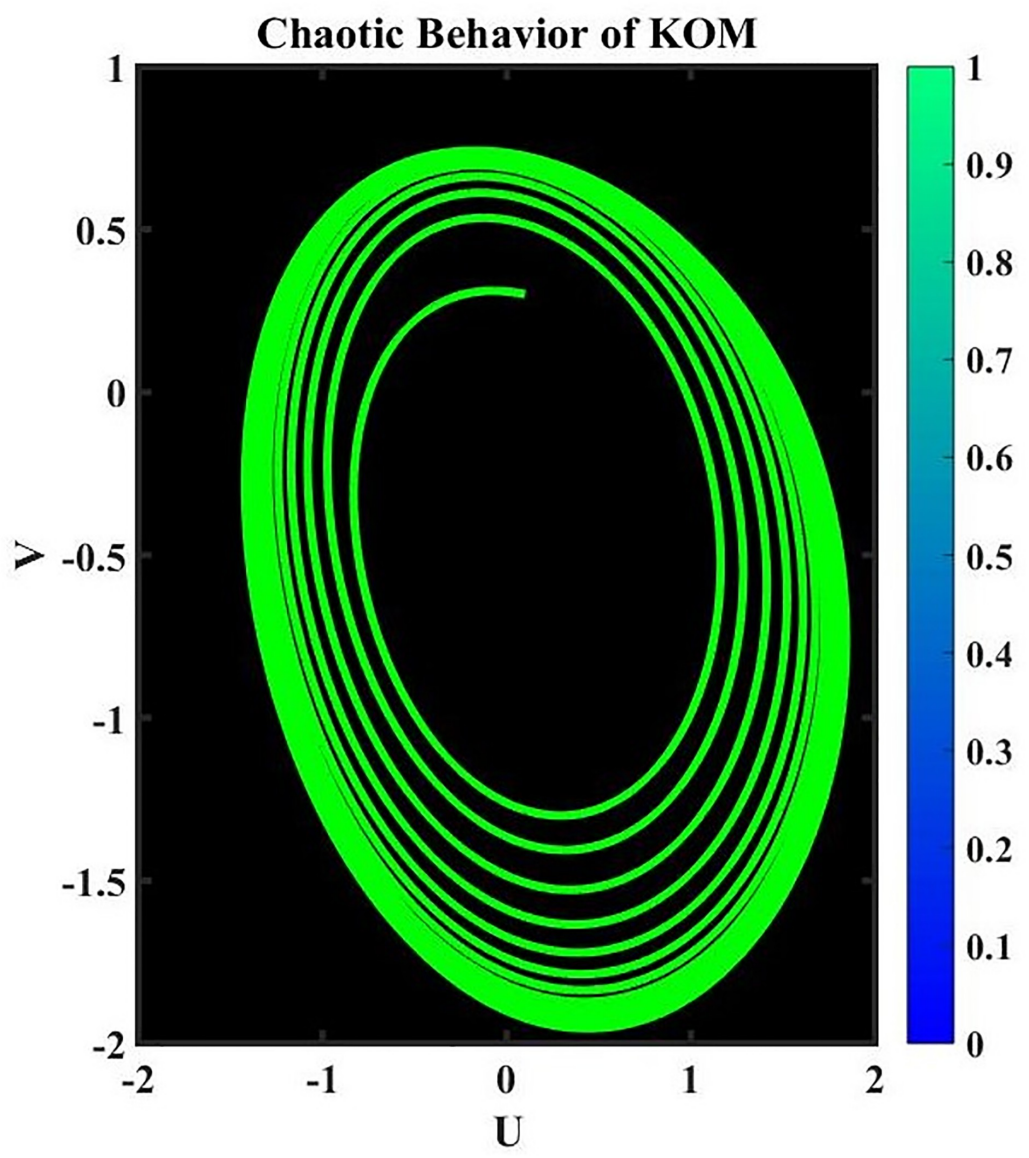
Physical depiction for chaotic behavior of (56), when *ε* = 3.3 & *ψ* = 1, and the suitable parametric values are *s* = 3.14, *q* = 2.35 & *L* = 3.12.

**Fig 23:**
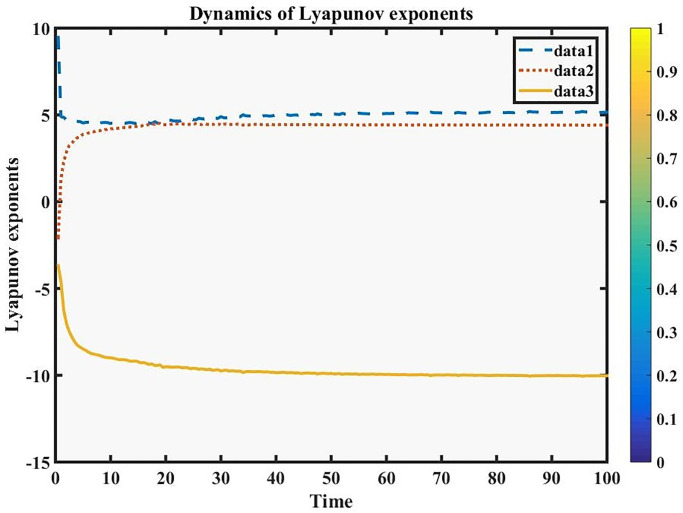
Physical depiction of Lyapunov exponent for the existence of chaos, under parametric values are data-1, data-2 and data-3, that is given in the following Table 2.

**Table 2:** (Data-1) represent the values of blue dashes, (Data-2) represent the values of red dots and (Data-3) represent the values of yellow line.

TIME	DATA-1	DATA-2	DATA-3
10.0000	4.509586	4.207012	-8.989843
20.0000	4.696497	4.434386	-9.507721
30.0000	4.906046	4.434904	-9.752216
40.0000	4.999213	4.421490	-9.849055
50.0000	5.023194	4.429611	-9.891405
60.0000	5.083570	4.422665	-9.951761
70.0000	5.115007	4.421844	-9.987259
80.0000	5.127086	4.417038	-9.998178
90.0000	5.139134	4.424619	-10.020664
100.0000	5.156810	4.409855	-10.025867

## Conclusions

In order to investigate the FCKOM, this study used the unified technique. This method was used to obtain novel soliton solutions, including trigonometric, rational, and hyperbolic ones. Using appropriate parameter values, 2D, density and 3D graphs were used to visualize the results. Additionally, the FCKOM equation’s perturbed and unperturbed dynamical systems both performed a phase illustration analysis. Bifurcation theory and chaos theory were also used to analyze the system’s behavior. The results of the research showed that the system was composed of both stable and unstable manifolds, bifurcation points and the onset of chaotic behavior. The observed soliton solutions and the analysis of the system dynamics establish a framework for future study in soliton dynamics, stability analysis and the investigation of nonlinear phenomena in related systems. They offer insightful information on the nonlinear behavior of the FCKOM. The conclusions of this study provide various directions for further investigation. The discovered soliton solutions’ stability and robustness to various perturbations and parameter changes can first be explored through additional research. It will be easier to use the solitons in real-world applications. Understanding soliton dynamics, bifurcations, and chaos offers insightful knowledge into a variety of physical, mathematical and engineering systems, providing potential for advancement in a variety of fields of science and technology.
